# Early Upper Palaeolithic marine mollusc exploitation at Riparo Bombrini (Balzi Rossi, Italy): shellfish consumption and ornament production

**DOI:** 10.1007/s12520-024-02148-5

**Published:** 2025-01-31

**Authors:** Silvia Gazzo, Emanuela Cristiani, Fabio Negrino, Julien Riel-Salvatore

**Affiliations:** 1 Department of Antiquities, Philosophy, History, University of Genoa, Genoa, Italy; 2 DANTE – Diet and ANcient TEchnology Laboratory, Department of Oral and Maxillo-Facial Sciences, Sapienza University of Rome, Rome, Italy; 3 Département d’anthropologie, Université de Montréal, Montréal, QC Canada

**Keywords:** Marine resources, Shell beads, Protoaurignacian, Early Upper Palaeolithic, *Homo sapiens*

## Abstract

This research explores the modes of exploitation of marine molluscs at Riparo Bombrini (Ventimiglia, north-west Italy) during the Protoaurignacian and the Early Aurignacian. Our results prove that Early Modern Humans who inhabited the rockshelter extensively exploited marine malacofauna for both dietary purposes and ornament production, offering new insights into human adaptation to coastal environments during the early phases of the Upper Palaeolithic along the Mediterranean coast. Combining taxonomy and taphonomy, we identified five main categories of shell remains within the assemblage: edible specimens, shell beads, non-worked ornamental shells, accidental introductions, and potential ornamental shells. A total of 91 perforated gastropods were recovered during the excavations of the Early Upper Palaeolithic layers. The ornament assemblage shows a certain richness in mollusc species, whose shells were collected dead from the beach. However, a preference for spherical and semi-spherical shells can be observed, highlighting the existence of trends in the selection of shell species for bead production. Use wear analysis demonstrates that some of the shell beads exhibit rounding and polishing around the rim of the perforation, implying that most of them arrived at the site as worn components, possibly forming part of more complex decorative combinations. Finally, the presence of both perforated and unperforated shells interpretable as raw material suggests that the rockshelter served as a “manufacturing site”, where shell ornaments were fabricated, discarded and replaced in new beadworks. This hypothesis is further supported by the presence of broken shell beads, interpretable as manufacturing errors or worn beads ready for replacement.

**Supplementary Information:**

The online version contains supplementary material available at 10.1007/s12520-024-02148-5.

## Introduction

Marine resources were widely exploited by hunter-gatherer groups across Europe during the Middle and Upper Palaeolithic. Among these resources, marine molluscs were used for various purposes: they played a role in the diet of both Neanderthals and Anatomically Modern Humans (AMHs), but they were also collected and used as raw material for tool and ornament production, thanks to their polycrystalline biomineral nature which gave them excellent mechanical properties (Barthelat et al. 2009; Liang et al. 2008).

In the last decades, the importance of marine resources during the Palaeolithic has begun to receive greater attention (e.g., Álvarez-Fernández 2010,2011,2015; Bicho and Esteves 2022; Colonese et al. 2011; Cortés-Sánchez et al. 2011). The systematic study of marine mollusc remains from Mediterranean sites has thus widened our knowledge about the ancestry of littoral resource exploitation and their potential as key resources across cycles of climatic changes and demographic crises. Coastal regions generally provide abundant and easily accessible marine food resources, which made these areas attractive to Palaeolithic human groups (e.g., Davies 2011; Marean 2011; Steele and Álvarez-Fernández 2011; Szabo and Amesbury 2011).

Until recently, evidence for the systematic exploitation of marine molluscs for dietary purposes was considered as one of the defining traits of the Upper Palaeolithic behavioural ‘revolution,’ interpreted as evidence of a progressive broadening of human diets. However, over the past two decades, there has been accumulating evidence that Neanderthals also widely exploited intertidal marine resources, showing that marine resource exploitation was not confined to AMHs (e.g., Colonese et al. 2011; Cortés-Sánchez et al. 2011; Gamble 1986; Fa 2008; Mellars and Tixier 1989; Stiner 1999; Stringer et al. 2008).

Indeed, molluscs have been exploited by humans since before Marine Isotope Stage 5 (MIS 5). The earliest evidence comes from the site of Pinnacle Point Cave 13B (PP13B), on the south coast of South Africa, dating to ~ 164 ka (± 12 ka) (Jerardino 2016; Jerardino and Marean 2010; Marean et al. 2007). In the Mediterranean area, solid evidence of the exploitation of marine resources (molluscs but also marine mammals, crustaceans, echinoderms and fishes) come from several Middle Palaeolithic sites attributed to neanderthals, such as Grotta dei Moscerini (Latium, Italy), Cueva de Los Aviones (Cartagena, Spain), Cueva Bajondillo (Málaga, Spain), and Cueva de Figueira Brava (Arrabida, Portugal) (Álvarez-Fernández 2015; Colonese et al. 2011; Stiner 1993, 1994; Villa et al. 2020).

In contrast, personal ornaments (including, but not limited to, shell beads) are commonly linked to the emergence of a complex symbolic behaviour. They first appeared during the Middle Stone Age (MSA) of Africa and around 100 kya in the Levant, becoming materials of pivotal importance for the debate on behavioural modernity. Moreover, since they are objects loaded with symbolic meaning, with limited geographic origins, shell beads are particularly suitable for studying past exchange networks and long-distance circulation routes (e.g., Bar-Yosef and Hayes 1989; Choyke 2013; Kuhn and Stiner 2006; Lock and Symes 1999; Newel et al. 1990; Peschaux 2017; Rigaud et al. 2015, 2018; Taborin 1993a, 2004; Vanhaeren 2010).

In Europe, several Mousterian contexts have yielded sporadic evidence of alleged symbolic objects, with cultural practices involving the joint use of shells and red ochre (e.g., Peresani et al. 2013; Zilhão et al. 2007, 2010).

The so-called “transitional” complexes are also marked by greater frequencies and diversity of ornaments, including the occasionally use and manufacturing of ornaments, such as perforated, grooved, and pigment-stained pendants (e.g., d’Errico et al. 2003a, b; Flas 2011; Hublin 2015; Moroni et al. 2013, 2018; Riel-Salvatore 2009; Škardla 2013; Zilhão 2006). However, systematic production of ornaments appears in Europe only with the Protoaurignacian and Early Aurignacian. The Early Upper Palaeolithic thus marked the florescence of formal ornament traditions, with personal ornaments becoming among the earliest behavioural indicators of human cognitive complexity, reflecting the ability to share coded information within and across human groups (e.g., Bar-Yosef et al. 2009; Borić and Cristiani 2019; d’Errico et al. 2009; Kuhn et al. 2001; Peresani et al. 2019; Stiner 1999, 2003; Taborin 2004; Vanhaeren and d’Errico 2001; Vanhaeren et al. 2013; White 1989). Personal ornaments also reveal possible intriguing information about Palaeolithic ethno-linguistic groups (Vanhaeren and d’Errico 2006), social and territorial organisation, interlinking exchange systems, as well as long-distance social networks (e.g., Álvarez Fernández 2001; Fritz and Simmonet 1996; Newell et al. 1990; Stiner 2003; Taborin 1993a, b; Vanhaeren and d’Errico 2003, 2005, 2006; Vanhaeren et al. 2004, 2013; White,^1989^).

This study examines the nature of marine mollusc exploitation during the Early Upper Palaeolithic, using the Protoaurignacian and the Early Aurignacian levels from Riparo Bombrini as a case study. It explores how the natural availability of these resources shaped hunter-gatherers’ food choices and raw material selection, shedding light on the adaptive strategies employed during the early dispersal of AMHs along the northwestern Mediterranean coast.

## Research history and site background

**Fig. 1:**
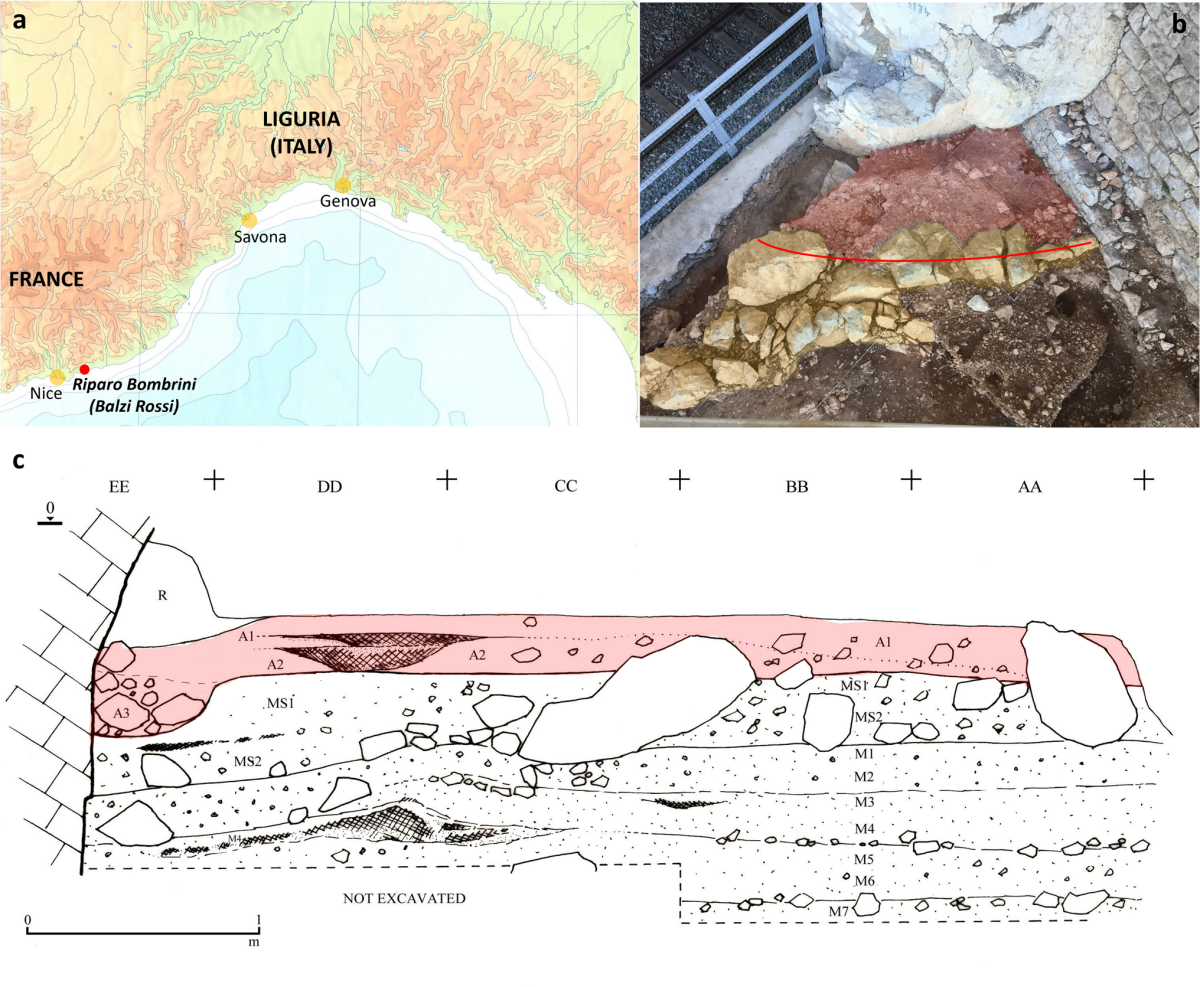
**a**) Location of Riparo Bombrini, in Liguria (north-west Italy); **b**) site contextualisation showing the large blocks of roof fall during a cold phase (yellow) and the internal area of the rockshelter (red). The red line shows the approximate position of the dripline; **c**) stratigraphic profile of Riparo Bombrini highlighting Protoaurignacian levels A1, A2 and A3. R indicates the reworked deposit. (Designed by F.N. and modified by S.G.)

Riparo Bombrini (43°47’1.63"N, 7°32’6.26"E)^1^ is a collapsed rockshelter that is part of the storied Balzi Rossi site complex, an iron-stained cliff formation of Upper Jurassic dolomitic limestone, in the Imperia province of the Liguria region (NW Italy) (Fig. 1a and b). The site contains rich Late Mousterian, Protoaurignacian and Early Aurignacian deposits (Holt et al. 2019).

First tested in 1938 (Cardini 1938), Riparo Bombrini was properly investigated using controlled recovery and documentation methods by G. Vicino in 1976 (Vicino 1984), ahead of the construction of a walkway built to facilitate access to the other sites of the Balzi Rossi complex. A quarter century later, excavations led by E. Formicola, B. Holt and F. Negrino resumed at the site, lasting from 2002 to 2005. From 2015 to 2022, renewed excavations at the site began under the direction of two of the authors (F.N. & J.R.S.) as part of larger efforts to investigate the Ligurian Palaeolithic (Bertola et al. 2013; Holt et al. 2019; Negrino et al. 2016, 2018; Negrino and Riel-Salvatore 2018; Negrino et al. 2023; Riel-Salvatore 2010; Riel Salvatore and Negrino 2018a, b; Riel-Salvatore et al. 2022, 2023). The most recent fieldwork at the site has applied new analytical methods to document the transition over a more extensive area. These include the large-scale implementation of a ZooMS program to identify faunal strategies, the use of photogrammetry and total station to document the site and the evolution of the excavation, as well as the use of cryptotephrochronology to resolve some outstanding chronological issues at the site (Hirniak et al. 2019; Martin-Moya et al. 2020; Pothier Bouchard et al. 2019, 2020; Vallerand 2021; Vallerand et al. 2024).

These three projects have made it possible to distinguish three macro-units of human occupation at Riparo Bombrini (Benazzi et al. 2015; Higham et al. 2014; Holt et al. 2019; Riel-Salvatore et al. 2013, 2022, 2023). At the very top of the sequence and limited to a narrow part of the excavated area, a residual deposit referable to the Early Aurignacian was identified only during the 1976 fieldwork and named A0. In contrast, the Protoaurignacian levels A1 and A2 were identified over the entire explored area, while level A3 was present only as a strip of relict deposits against the site’s back wall that contained almost no artefacts (Fig. 1c). AMS dates, stratigraphic and sedimentological evidence indicate that the Protoaurignacian at Bombrini dates to around 41-36.5 ka cal. BP (Benazzi et al. 2015; Holt et al. 2019).

The second macro-unit is clearly separated from A3 and A2 by an erosional paraconformity and comprises the ‘semi-sterile’ Final Mousterian deposits (divided into sub-units MS1-2) thought to have accumulated between ca. 43 − 42 ka cal. BP. Less abundant traces of human activity and the presence of carnivore coprolites point to the sporadic but patterned use of the shelter by hominins during this period (Riel-Salvatore et al. 2022; Vallerand et al. 2024).

The third macro-unit is constituted by levels M1-7, which accumulated between ca. 45 − 43 ka cal. BP; they form a reddish clayey loam deposit 70 cm thick that yielded rich Late Mousterian assemblages (especially in M3 and M5), and several large discrete concentrations of charcoal in M4.

## Materials and methods

This study employs a multidisciplinary approach that combines taxonomy, marine ecology, taphonomy, as well as technological and use wear analyses.

At Riparo Bombrini, shell assemblage comprises (1) edible molluscs, including large gastropods and bivalves and (2) non-edible molluscs, which include accidental introductions, shell beads and non-worked ‘ornamental shells’.

Marine mollusc remains from the Early Aurignacian (A0) and Protoaurignacian (A1, A1-A2, A2 and A3) levels of Riparo Bombrini were analysed. These materials were collected over multiple field campaigns (1976, 2002–2005, 2015–2019, and 2022), during which the same recovery methods were employed.

Specimens greater than 2 cm in maximum dimension were collected manually and individually plotted in three dimensions during the excavation. Smaller remains were recovered after wet sediment sieving through 2 mm mesh and their provenance (layer and square) was systematically registered.

Taxonomic classification was carried out using modern comparative collection (S.G.) supported by specialised bibliographies on Mediterranean malacology (e.g., Cossignani and Ardovini 2011; Doneddu and Trainito 2010). Several online atlases such as the *General Shell Portal* (http://www.idscaro.net/sci/) were also consulted to ensure the accuracy in the identification of the mollusc species. For consistency, the nomenclature follows the updated datasets available online on the *World Register of Marine Species* (WoRMS) (www.marinespecies.org*).* Finally, a consideration of the configuration and distribution of the regional biotopes particular to each shell species was performed using the *Paleobiology Database* (https://paleobiodb.org/#/*)* to investigate the modern and paleogeographic locations of the identified taxa.

Malacological remains were quantified using standard zooarchaeological methods: to estimate the abundance of each species, we calculated the Number of Remains (NR), the Number of Identified Specimens (NISP) and the Minimum Number of Individuals (MNI). MNI values for archaeological molluscan assemblages are most often calculated by counting the frequency of a selected range of Non-Repetitive Elements (NREs): whole or semi-whole valves and hinges or umbos for bivalves, and whole or semi-whole individuals and apices for gastropods (Allen 2012; Ballbè 2005; Chicoine and Rojas 2013; Claassen 1998; Mannino and Thomas 2001; Mason et al. 1998; Ono and Clark 2012; Poteate and Fitzpatrick 2013; Seeto et al. 2012). Current MNI calculation protocols, however, have the potential to consistently underestimate the relative abundance of taxa (Gutiérrez-Zugasti 2011; Harris et al. 2015). We therefore incorporated a wider range of NREs, reducing the inherent bias towards particular shell forms. Gastropod NREs used for MNI calculation are as follows: (1) spire, (2) columella, (3) outer lip, (4) aperture, (5) umbilicus, and (6) operculum. The most frequent NRE for each gastropod taxon is the MNI. Bivalve NREs are (1) umbo, (2) hinge teeth, (3) anterior and (4) posterior portion of the hinge. The most frequently occurring NRE for the valve side with the highest count is the MNI. Scaphopod NREs are (1) apical, (2) mesial and (3) basal fragments.

Zooarchaeological and taphonomic investigations were carried out on the mollusc assemblage (NR 2645; NISP 2638), including both complete and fragmented specimens. All the shells presenting anthropic perforation (NISP 91) were analysed at the DANTE-Diet and Ancient Technology Laboratory of Sapienza University of Rome to characterise their use as ornaments.

Using a digital calliper, two general categories of biometric and morphometric data were also recorded: (1) the dimensions of the shells (maximum length, width, and width of the aperture) (Allen 2017); and (2) the dimensions of the anthropogenic perforations in shell beads (maximum length and width).

Taphonomic analysis was performed at low magnifications using a Leica S6D Greenough stereomicroscope (magnification ranging from 0.75x to 70x).

The Fragmentation Index (F.I.) was calculated for each taxonomic group and layer (based on the MNI/NISP ratio). For a better characterization of the general state of preservation of the assemblage, we recognised three different degrees of shell completeness that depended on the percentage of shell that had been preserved: (1) fragmented specimens (less than 50% is preserved); (2) partially complete specimens (50–90% is preserved); and (3) complete specimens (between 90 and 100%, with all the diagnostic parts of the shell preserved).

The techno-functional investigation of the ornaments was carried out according to previous research on the production and use of shell and osseous beads from prehistoric contexts (e.g., Arrighi et al. 2019; Bertolini et al. 2016; Bonnardin 2008; Bouzouggar et al. 2007; Cristiani and Borić 2012; d’Errico et al. 1993; d’Errico and Vanhaeren 2002; Taborin 1993a, b; Tátá et al. 2014; Vanhaeren and d’Errico 2001, 2003, 2005; Vanhaeren et al. 2013; White 1989).

In addition to the available literature, we performed targeted experimental activities on modern *Homalopoma sanguineum* specimens to test technological interpretations of the archaeological ornaments from Riparo Bombrini.

The location of the anthropogenic perforations was documented based on the criteria developed by Taborin (1993a, b) for mapping the position of holes on the shells. Perforation techniques were identified through the observation of hole contour, morphology, and microscopic damage (d’Errico 1993). Use wear localization on the rims of the holes was categorized into four areas: A and B correspond to the margins located near the aperture, while C and D are the closest to the apex of the shell (Fig. 8b). Variables such as change in colour, faceting, deformation of the outline, striations, invasiveness of the wear were also used to interpret the use of ornaments, according to published literature (Cristiani et al. 2014; Gravel-Miguel et al. 2022).

Technological and use wear modifications were identified using a Zeiss Axio Zoom V16 digital stereomicroscope equipped with a Plan-NeoFluar Z1x/0.25 FWD objective with magnifications ranging from 10x to 168x. Some perforated shells, use wear traces were selected and observed at high magnification, using a ZEISS AxioScope A1 metallographic microscope with magnification ranging from100x to 500x.

## Results

### Taxonomy

The total NR for which a secure stratigraphic position could be ascertained on the basis of their labels is 2645 (NISP 2638; MNI 601) (Table 1).

We identified a total of 83 taxa comprising 69 gastropods and 12 bivalves. Only one taxon (*Antalis* sp.) has been identified for scaphopods. Undetermined shell remains, for which the class cannot be identified, were treated as a distinct taxon.

**Fig. 2:**
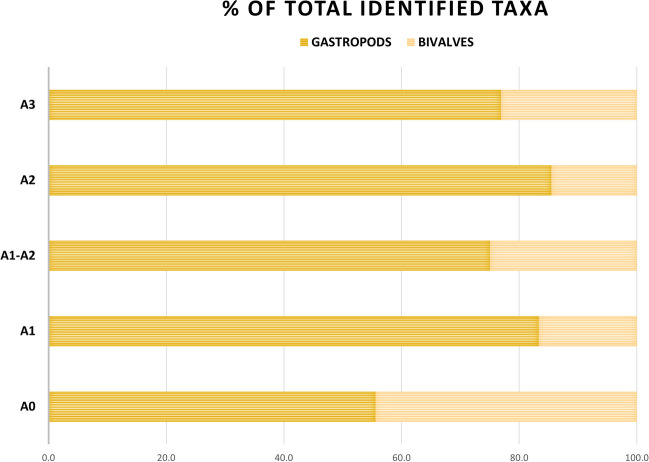
Comparison of taxonomic richness (N of identified taxa) for marine gastropods and bivalves across the investigated levels

From a taxonomic point of view, the studied assemblage does not differ much from the natural frequency of species observed for living marine communities, which generally consist of 80% gastropods and 20% bivalves (Sabelli 1980). The proportions related to taxonomic richness are thus very close to the worldwide standard for living mollusc communities: gastropods representing 83.1% of the entire assemblage, whereas bivalves represent 14.5%. These percentages are similar across all investigated levels, suggesting that the collection of marine molluscan fauna was constrained by the taxonomic richness inherent to the ecological diversity of the marine environment (Fig. 2). An exception is observed at level A0, where the collection was almost exclusively focused on bivalves of the species *Mytilus galloprovincialis*.

Levels A1 (NISP 1540), A0 (NISP 545), and A2 (NISP 487) exhibit higher concentrations of mollusc remains, whereas only a small number was recovered from A3 (NISP 52). Moreover, originating from the composite unit A1-A2, established in the uncertainty of confidently assigning findings to either level, only a scant number of specimens were additionally unearthed (NISP 14).

Overall, the edible mussel *M. galloprovincialis* is the most abundant species (NISP 1956) representing 74.1% of the total NISP, followed by small gastropods with no nutritional value such as *H. sanguineum* (NISP 115), *Bittium* sp. (NISP 80) and *Tritia neritea* (NISP 52). The remaining 16% is made up of different species of sea snails (e.g., *Cerithium vulgatum*, *Jujubinus* cf. *exasperatus*,* Melarhaphe neritoides*, *Ocenebra edwardsii*), limpets (*Patella* sp., *Patella rustica*, *Patella* cfr. *rustica*), clams (e.g., *Callista chione*, *Chamelea* cf. *striatula*, *Glycymeris* sp.) and scallops (e.g., *Pecten jacobaeus*, *Pecten* sp.).

The presence of fragments of *C. chione* in levels A1 and A1-A2 is discussed in the supplementary material (Supplementary Information 1; Fig. S1).

Gastropods are very well documented in all levels, with level A2 standing out by yielding more than half (51.5%) of all identified gastropod remains. Notably, *H. sanguineum* is well-documented in levels A1 and A2, comprising a substantial portion (20.1%) of the gastropod sample.

Among nassarids (20.4%), the most represented species are *Tritia neritea* (previously known as *Cyclope*) and *Tritia incrassata.* Other species (e.g., *Tritia* cfr. *pellucida*, *Tritia gibbosula*, and *Tritia corniculum*) are only sporadically documented in the studied assemblages.

All bivalves were found as single valves or fragments of them. *M. galloprovincialis* overwhelmingly dominates their composition (94.9%), sometimes appearing as a homogeneous cluster that includes very altered fragments embedded in concretions. This species is mostly documented in levels A0 and A1, with its abundance decreasing in levels A2 and A3.

Finally, a total of 71 non-*Mytilus* bivalve remains (scallops and clams) were identified, mainly in level A2.

**Table 1:** Number of remains (NR), including undetermined remains, number of identified specimens (NISP), and Minimum Number of individuals (MNI) for each level. Specimens with anthropic perforations are reported within parentheses

TAXA	A0		A1		A1-A2		A2		A3		NISP	% NISP
	NISP	MNI	NISP	MNI	NISP	MNI	NISP	MNI	NISP	MNI		
**GASTROPODA**												
**Nassariidae**												
*Tritia* cfr. *pellucida*	-	-	3 (2)	3	-	-	1 (1)	1	-	-	4	0.15
*Tritia* cfr. *neritea*	-	-	1	1	-	-	8 (4)	8	-	-	9	0.34
*Tritia neritea* (Linnaeus, 1758)	-	-	26 (18)	26	-	-	26 (14)	23	3 (3)	3	55	2.08
*Tritia* sp. (*neritea*/*pellucida*)	-	-	5	5	-	-	6	6	-	-	11	0.42
*Tritia incrassata* (Strøm, 1768)	-	-	14 (3)	14	-	-	1	1	-	-	15	0.57
*Tritia gibbosula* (Linnaeus, 1758)	-	-	2 (1)	2	-	-	4 (1)	4	-	-	6	0.23
*Tritia corniculum* (Olivi, 1792)	-	-	4	4	-	-	2	2	-	-	6	0.23
*Tritia nitida* (Jeffreys, 1867)	-	-	2 (2)	2	-	-	-	-	-	-	2	0.08
*Tritia mutabilis* (Linnaeus, 1758)	-	-	2 (1)	2	-	-	-	-	-	-	2	0.08
*Tritia* cfr. *cuvierii*	-	-	-	-	-	-	1	1	-	-	1	0.04
Nassariidae	-	-	-	-	-	-	4 (1)	2	2	1	6	0.23
**Cerithiidae**												
*Bittium reticulatum* (da Costa, 1778)	-	-	1	1	1	1	16	15	6	6	24	0.91
*Bittium* cfr. *reticulatum*	-	-	1	1	-	-	2	2	-	-	3	0.11
*Bittium latreillii* (Payraudeau, 1826)	-	-	4	4	-	-	8	8	-	-	12	0.45
*Bittium* cfr. *latreillii*	-	-	-	-	2	2	2	2	-	-	4	0.15
*Bittium lactescens* (Jeffreys, 1867)	-	-	3	3	-	-	1	1	2	2	6	0.23
*Bittium* cfr. *lactescens*	-	-	4	4	1	1	-	-	-	-	5	0.19
*Bittium lacteum* (R. A. Philippi, 1836)	-	-	1	1	-	-	-	-	-	-	1	0.04
*Bittium* cfr. *lacteum*	-	-	2	2	-	-	-	-	-	-	2	0.08
*Bittium* cfr. *incile*	-	-	1	1	-	-	-	-	-	-	1	0.04
*Bittium* sp.	-	-	23	19	4	3	35	31	18	17	80	3.02
*Cerithium vulgatum* Bruguière 1792	1	1	2	2	-	-	4	4	-	-	7	0.26
*Cerithium* cfr. *vulgatum*	-	-	2	2	-	-	1	1	-	-	3	0.11
*Cerithium lividulum* Risso, 1826	-	-	1	1	-	-	-	-	-	-	1	0.04
*Cerithium* sp.	-	-	2	1	-	-	2	1	-	-	4	0.15
Cerithiidae	-	-	-	-	1	1	9	3	-	-	10	0.38
**Muricidae**												
*Ocenebra edwardsii* (Payraudeau, 1826)	-	-	8 (4)	8	-	-	3 (2)	3	1	1	12	0.45
*Ocenebra* cfr. *edwardsii*	-	-	-	-	-	-	1	1	-	-	1	0.04
*Ocinebrina aciculata* (Lamarck, 1822)	-	-	2	2	-	-	1	1	-	-	3	0.11
*Ocinebrina* cfr. *aciculata*	-	-	-	-	-	-	1	1	-	-	1	0.04
*Ocinebrina* sp.	-	-	3	3	-	-	1	1	-	-	4	0.15
Muricidae	-	-	2	2	-	-	2	1	1	1	5	0.19
**Trochidae**												
*Jujubinus* cfr. *exasperatus*	1	1	2	2	-	-	10	10	1	1	14	0.53
*Gibbula* sp.	-	-	3	2	-	-	7	3	2	1	12	0.45
*Phorcus turbinatus* (Born, 1778)	-	-	2 (1)	2	-	-	-	-	-	-	2	0.08
*Phorcus* sp.	1	1	3	3	-	-	1	1	-	-	5	0.19
*Steromphala adansonii* (Payraudeau, 1826)	-	-	1	1	-	-	-	-	-	-	1	0.04
*Clanculus jussieui* (Payraudeau, 1826)	-	-	-	-	-	-	2	2	-	-	2	0.08
*Clanculus corallinus* (Gmelin, 1791)	-	-	1	1	-	-	2 (1)	2	-	-	3	0.11
*Clanculus* cfr. *cruciatus*	-	-	-	-	-	-	1 (1)	1	-	-	1	0.04
Trochidae	-	-	1	1	-	-	3	2	-	-	4	0.15
**Littorinidae**												
*Melarhaphe neritoides* (Linnaeus, 1758)	-	-	5	5	1	1	4	4	-	-	10	0.38
*Littorina obtusata* s.l.	-	-	1	1	-	-	1 (1)	1	-	-	2	0.08
*Littorina saxatilis* (Olivi, 1792)	-	-	-	-	-	-	2	2	-	-	2	0.08
**Colloniidae**												
*Homalopoma sanguineum* (Linnaeus, 1758)	1	1	44 (10)	42	1	1	64 (15)	60	5	5	115	4.35
**Calliostomatidae**												
*Calliostoma zizyphinum* (Linnaeus, 1758)	-	-	2	2	-	-	-	-	-	-	2	0.08
*Calliostoma* sp.	-	-	1	1	-	-	2	2	-	-	3	0.11
**Triviidae**												
*Trivia* sp.	-	-	3 (1)	3	-	-	2	2	-	-	5	0.19
**Turbinidae**												
*Bolma rugosa* (Linnaeus, 1767)	-	-	1	1	-	-	3	3	-	-	4	0.15
**Columbellidae**												
*Columbella rustica* (Linnaeus, 1758)	-	-	-	-	-	-	1 (1)	1	-	-	1	0.04
*Mitrella gervillii* (Payraudeau, 1826)	-	-	3	3	-	-	2	2		-	5	0.19
*Mitrella* cfr. *gervillii*	-	-	-	-	-	-	1	1	-	-	1	0.04
*Mitrella* sp.	-	-	1	1	-	-	-	-	-	-	1	0.04
Columbellidae	-	-	-	-	-	-	1	1	-	-	1	0.04
**Naticidae**												
*Notocochlis dillwynii* (Payraudeau, 1826)	-	-	-	-	-	-	1	1	-	-	1	0.04
*Euspira macilenta* (R. A. Philippi, 1844)	-	-	1 (1)	1	-	-	1	1	-	-	2	0.08
**Turritellidae**												
*Turritellinella tricarinata* (Brocchi, 1814)	-	-	6 (1)	6	-	-	4 (1)	4	-	-	10	0.38
**Rissoidae**												
*Alvania mamillata* Risso, 1826	-	-	-	-	-	-	1	1	-	-	1	0.04
*Alvania* sp.	-	-	-	-	-	-	2	2	-	-	2	0.08
**Mitridae**												
*Episcomitra cornicula* (Linnaeus, 1758)	-	-	-	-	-	-	2	2	-	-	2	0.08
**Vermetidae**												
*Vermetus* sp.	-	-	-	-	-	-	2	2	-	-	2	0.08
**Aporrhaidae**												
*Aporrhais pespelecani* (Linnaeus, 1758)	-	-	1	1	-	-	1	1	-	-	2	0.08
**Patellidae**												
*Patella rustica* Linnaeus, 1758	-	-	1	1	-	-	-	-	-	-	1	0.04
*Patella* cfr. *rustica*	2	2	-	-	-	-	-	-	-	-	2	0.08
*Patella* sp.	-	-	3	2	-	-	1	1	-	-	4	0.15
**Coralliophilidae**												
*Coralliophila meyendorffii* (Calcara, 1845)	-	-	1	1	-	-	-	-	-	-	1	0.04
**Rissoinidae**												
*Rissoina bruguieri* (Payraudeau, 1826)	-	-	-	-	-	-	1	1	-	-	1	0.04
**Conidae**												
*Conus* sp.	-	-	2	2	-	-	-	-	-	-	2	0.08
**Undetermined**	-	-	10	1	-	-	28	5	-	-	38	1.44
**TOTAL GASTROPODA**	**6**	**6**	**220**	**201**	**11**	**10**	**295**	**245**	**41**	**38**	**573**	**21.66**
**SCAPHOPODA**												
**Dentaliidae**												
*Antalis* sp.	-	-	-	-	-	-	3	1	1	1	4	0.15
**BIVALVIA**												
**Pectinidae**												
*Pecten jacobaeus* (Linnaeus, 1758)	2	1	-	-	-	-	7	1	-	-	9	0.34
*Pecten* cfr. *jacobaeus*	-	-	2	1	-	-	1	1	-	-	3	0.11
*Pecten* sp.	-	-	6	1	-	-	9	1	-	-	15	0.57
Pectinidae	-	-	2	1	-	-	10	1	-	-	12	0.45
**Cardiidae**												
*Acanthocardia* sp.	1	1	2	2	-	-	-	-	-	-	3	0.11
Cardiidae	-	-	3	1	-	-	11	1	2	1	16	0.60
**Veneridae**												
*Callista chione* (Linnaeus, 1758)	-	-	3	2	1	1	-	-	-	-	4	0.15
*Chamelea *cfr.* striatula*	1	1	1	1	-	-	-	-	-	-	2	0.08
**Glycymerididae**												
*Glycymeris* sp.	-	-	-	-	1	1	3	1	-	-	4	0.15
**Noetiidae**												
*Striarca lactea* (Linnaeus, 1758)	-	-	2	2	-	-	1	1	-	-	3	0.11
**Mytilidae**												
*Mytilus galloprovincialis* (Lamarck, 1819)	535	17	1287	46	-	-	131	4	3	1	1956	73.95
**Undetermined**	-	-	12	1	1	1	16	2	5	1	34	1.29
**TOTAL BIVALVIA**	**539**	**20**	**1320**	**58**	**3**	**3**	**189**	**13**	**10**	**3**	**2061**	**77.92**
**TOTAL NISP**	**545**	**26**	**1540**	**259**	**14**	**13**	**487**	**259**	**52**	**42**	**2638**	99.74
**Undetermined**	-	-	2	-	-	-	5	-	-	-	**7**	0.26
**GRAND TOTAL (NR)**	**545**	**-**	**1542**	**-**	**14**	**-**	**492**	**-**	**52**	**-**	**2645**	**100**

### Ecological aspects

During the period under consideration, lowered sea levels gave rise to an open plain in front of the Balzi Rossi cliffs, as proved by the presence of an equid species (likely *Equus ferus*) and rhino in level A2 (Holt et al. 2019). However, the Balzi Rossi complex has never been far from the shore due to the high topographic relief of the coast (Stiner 1999). The constant close proximity of Riparo Bombrini to the Tyrrhenian coastline allowed the ancient communities of the site to gather a wide range of molluscan species.

**Fig. 3:**
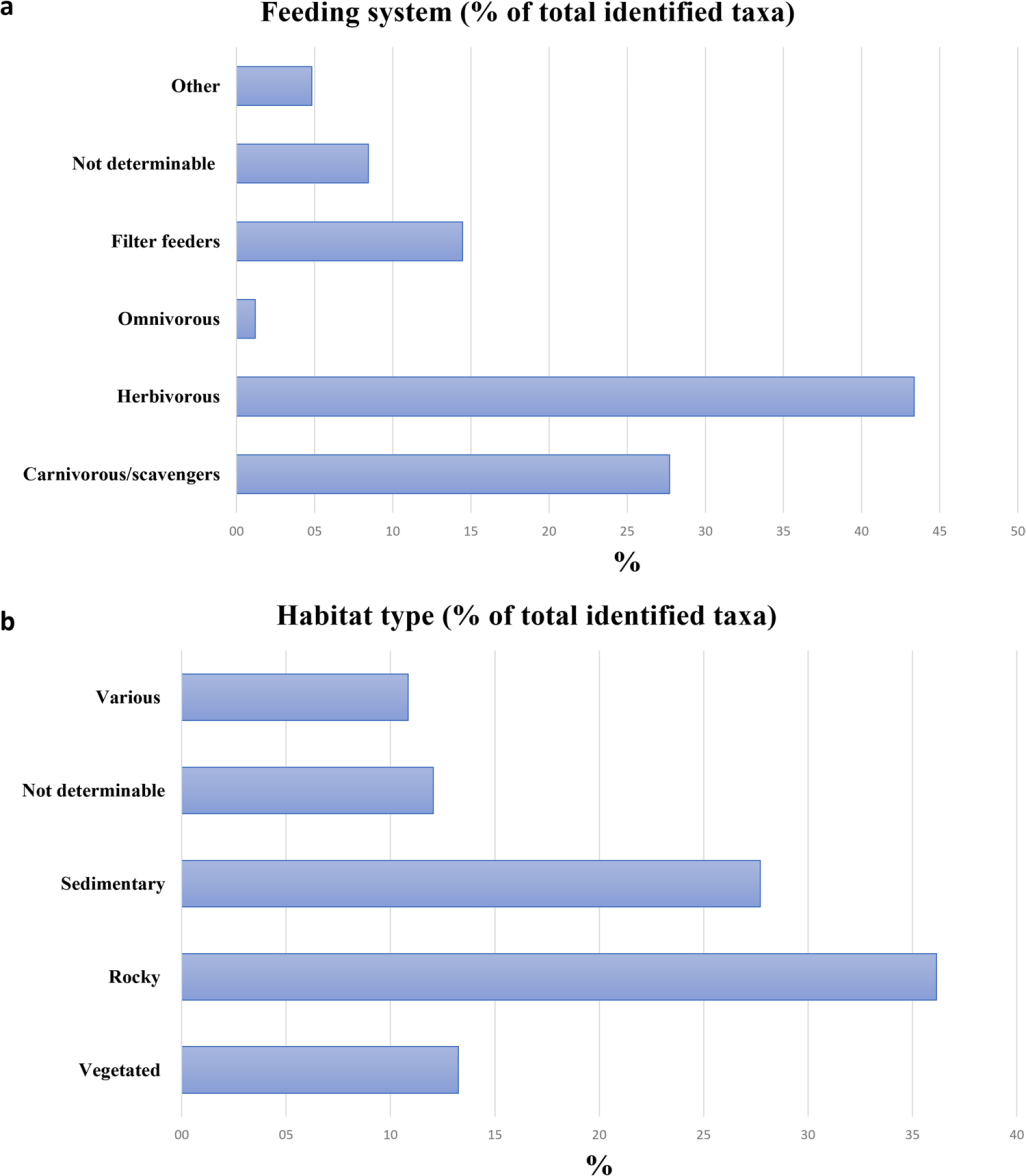
% of feeding systems (**a**) and habitat types (**b**) associated with the identified taxa

To gain insight into the ecology of molluscan communities during the Protoaurignacian, we grouped the identified taxa by system of feeding (Fig. 3a) and habitat type (Fig. 3b), providing information about the different ecological niches available to exploitation.

The marine assemblage reflects the exploitation of diverse marine environments, mainly including rocky shores, vegetated environments and sandy beaches that were probably tucked between the rocky cliffs of the coast.

Herbivorous and detritus feeders typical of vegetated marine habitats are dominant, indicating the presence of extensive meadows of *Posidonia oceanica* in the area surrounding the site. A similar environment can be observed today on the seabed of the Marine Reserve of Mortola (Imperia, Italy), where *Posidonia* grasslands extend from the Balzi Rossi complex to Latte. Gastropods of the genus *Bittium* are very common epiphytic grazers living in vegetated marine environments. Among them, *Bittium latreillii* represents a quantitatively dominant fraction of the whole fauna associated with *P. oceanica* leaves (Russo and Terlizzi 1998).

The intertidal zone was characterised by rocky shores, as indicated by the presence of edible gastropods typically found at different degrees of wave exposure (*Phorcus* spp., *Patella* spp., *Bolma Rugosa*) and mussels (*M. galloprovincialis*), which live bissally attached to rocks from the mid to low intertidal zone. *M. neritoides* thrives in dense populations along rocky shores, sometimes exceeding densities of 6000 individuals per square metre. This species occupies a prominent ecological niche as one of the dominant gastropods in the high-shore area (Fa 1998). *H. sanguineum* typically inhabit rocky bottoms, forming large colonies (Poppe and Goto 1991), but they are most abundant in shallow underwater caves and coral habitats (Dantart and Luque 1994; Radolović et al. 2015).

Most of the non-*Mytilus* bivalves are typically associated with soft fans of gravel or sand, implying that soft bottoms were locally present at some water depth, as suggested by the presence of *P. jacobaeus*, which inhabits coastal waters ranging from 25 to 100 m in depth, and *C. chione* (1–180 m depth) (Villa et al. 2020).

*T. neritea* is a euryhaline species (Boulhic and Tardy 1986) that inhabits tide- and river-influenced sandy and muddy substrates. It is commonly found in high densities in brackish lagoons, estuaries, and salt marshes (Boissin et al. 2020).

Contrary to *T. neritea.*, *T. pellucida*, which belongs to the biocenosis of the superficial muddy sands and prefers sheltered waters, only tolerates salt waters (0–2.5 m deep).

Boring and encrusting organisms also serve as important environmental indicators. For instance, the presence of boring sponges and bryozoans suggests a water salinity exceeding 15 ppt (Claassen 1998). Additionally, barnacles (*Balanus*), which are known to colonise stones, rocks, and shells, are virtually absent in pre-Holocene contexts since they are associated with warm waters. Their absence within the analysed assemblage may thus be considered as a marker of cold waters (Álvarez-Fernández 2013).

Information about cold-water species currently absent in the Mediterranean (i.e., *Littorina obtusata*/*Littorina fabalis* and *Littorina saxatilis* are given as supplementary material (Supplementary Information 2; Fig. S2).

### Taphonomy

#### Natural pre-depositional alterations

**Fig. 4:**
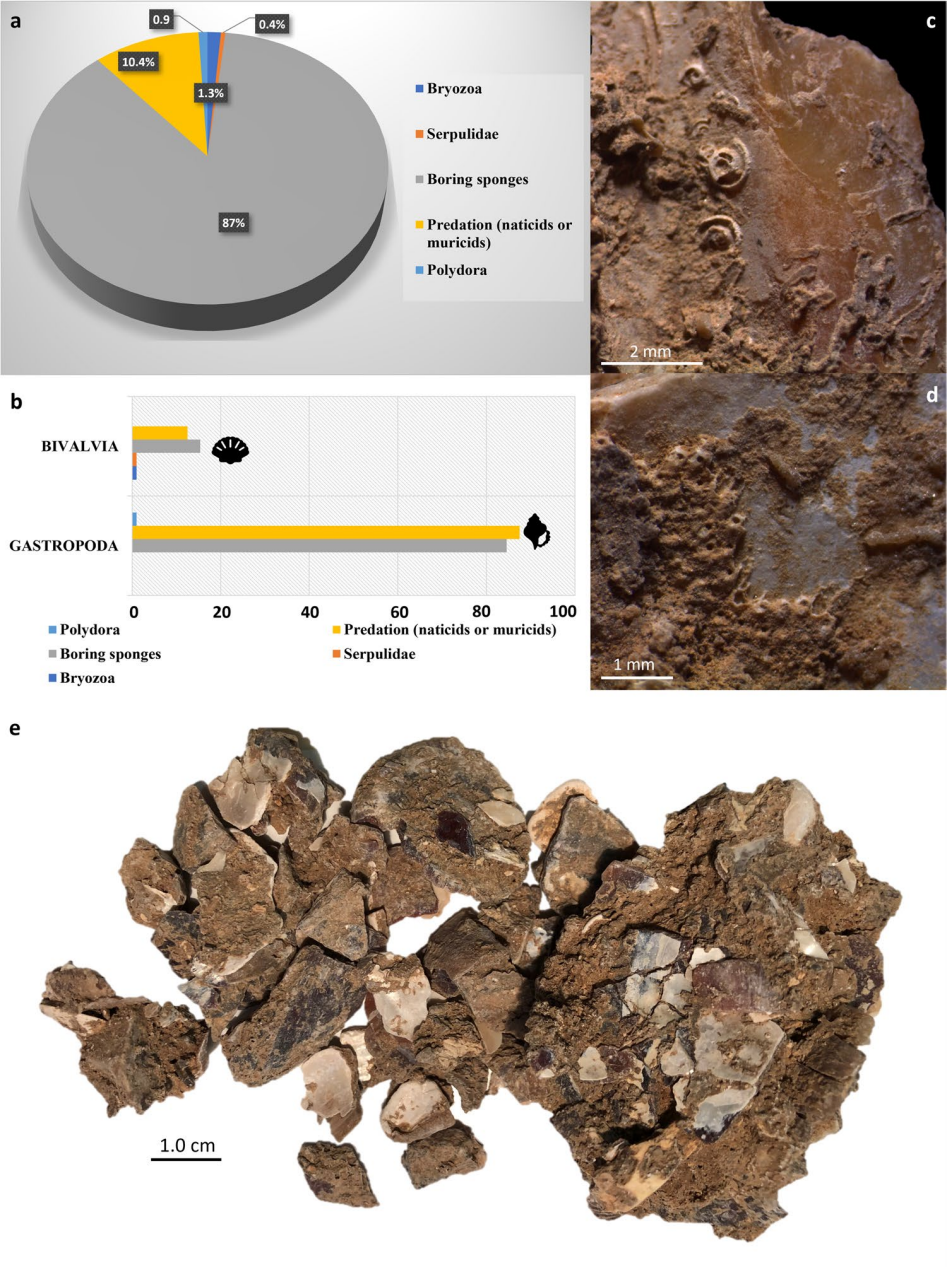
**a**) % of remains affected by pre-depositional alterations related to the marine environment (calculated on the NISP); **b**) % of bivalves and gastropods affected by pre-depositional alterations related to the marine environment (calculated on the NISP); **c**) fragment of *P. jacobaeus* affected by serpulids in the internal part of the valve (from level A1); **d**) *M. galloprovincialis* affected by bryozoans in the internal part of the valve (from level A0); **e**) fragments of *M. galloprovincialis* from level A1, some of which are enclosed within concretions

Natural pre-depositional alterations serve as indicators of peri- and post-mortem exposure to a marine environment. These alterations include abrasion resulting from an active beach environment (i.e., surface wear and polish resulting from beach rolling), predation by carnivorous snails (primarily boring gastropods), bioerosion and epizootic encrustation (Fig. 4a).

Micro-pits ascribed to the action of boring sponges occur in association with an overall beach-polished shell surface, suggesting prolonged exposure of shells to taphonomically active environments. A total of 201 specimens (7.6%) exhibit traces of clionid-like borings. These organisms typically produce small, rounded holes and rarely pierce the shell structure from one side to the other. Traces of boring sponges are predominantly observed on gastropods (NISP 170; 84.6%), while they are less common on bivalves (NISP 31; 15.4%) (Fig. 4b).

Elongated tunnels produced by shell-boring polychaetes (possibly *Polydora* sp.) were observed on *H. sanguineum* (NISP 1) and *T. corniculum* (NISP 1) from levels A2 and A1. Serpulids were found on the internal surface of one *P. jacobaeus* fragment (level A1), indicating that the valve was collected after the death of the animal (Fig. 4c).

As sessile organisms, bryozoans (Bryozoa) are among the most common components of encrusting communities on hard substrates. They were found on *Mytilus* shell fragments (NISP 3) from level A0 (Fig. 4d). Bryozoans were identified on both the internal and external surfaces of the valves, implying that at least some of the mussels were collected post-mortem, possibly becoming incorporated within colonies of living specimens.

**Fig. 5:**
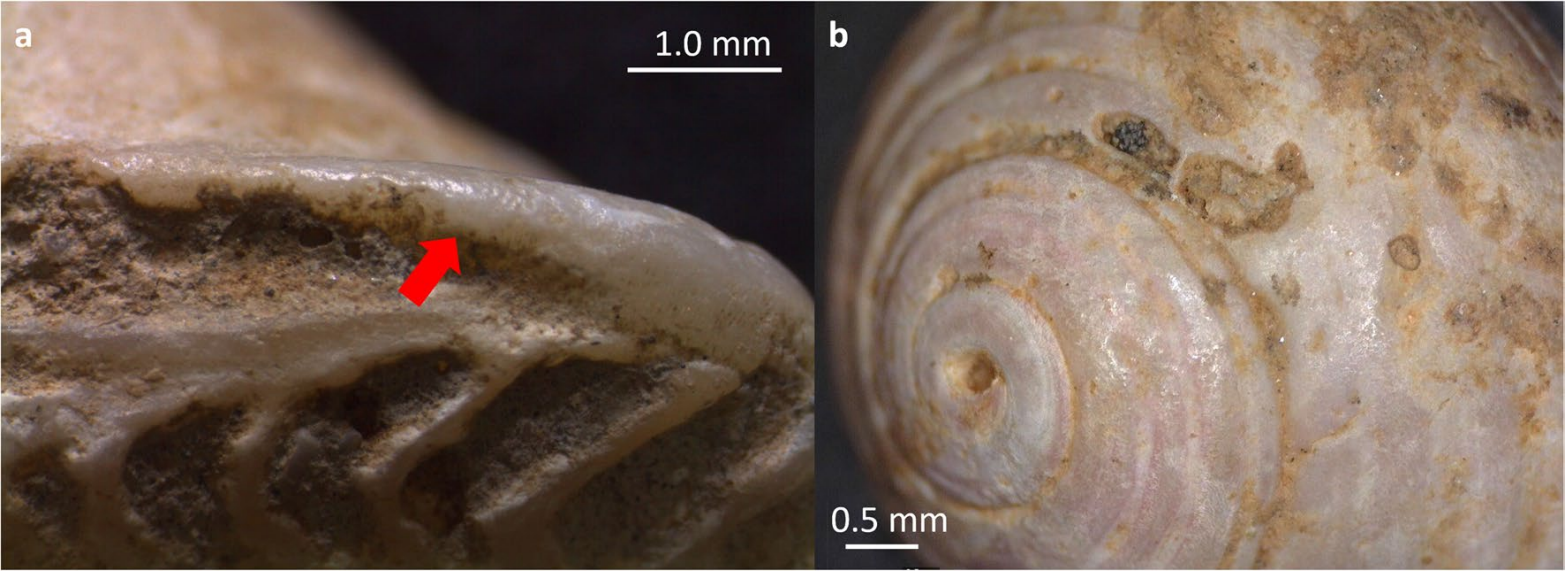
Marine abrasion. **a**) The red arrow highlights an intense abrasion near the hinge of a valve of *Glycymeris* sp. (from level A1-A2); **b**) homogeneous abrasion across the entire surface of a *H. sanguineum* shell (from level A2)

Our observations reveal beach polish induced by wave action and abrasion caused by sedimentary particles on both gastropods and bivalves across all the investigated levels, indicating varying degrees of abrasion intensity due to moderately energetic nearshore environments (Fig. 5). A total of 71 specimens (2.7%) exhibit perforations related to beach weathering. Natural perforations mostly affect specimens of the genus *Bittium* (NISP 41; 57.8%), whereas they are virtually absent on bivalves. Moreover, regular holes drilled by carnivorous gastropods, such as naticid or muricid predatory snails, were mainly detected on *Bittium* shells (NISP 3; 30%) and *M. galloprovincialis* (NISP 3; 30%).

#### Post-depositional transformations

A general overview of these alterations is provided in the supplementary material (Supplementary Information 3; Fig. S3a).

##### Fragmentation

Fragmentation is mainly associated with post-depositional taphonomic processes (e.g., trampling, sediment compaction, processes of sediment reworking), which probably led to further crushing during excavation and curation activities (Claassen 1998).

The F.I. (Supplementary Information 4; Table S4) reveals an excellent overall preservation, except for bivalves, which are virtually all (99.8%) fragmented. On the contrary, only 26.2% of the gastropods are fragmented, while the remaining gastropod shells are either complete (42.9%) or partially complete (30.9%). The low degree of fragmentation observed for gastropods may be attributed to both their small size and the natural resistance of their shell structure, which in many cases comprises a nacreous layer. On the other hand, bivalves tend to be inherently fragile and susceptible to fragmentation, given the physical structure of their valves and their vulnerability to trampling (Supplementary Information 3; Fig. S3b) (Stiner 2010).

Fragmentation affects all levels similarly; however, the highest percentage of fragmentation is observed in level A0, where 50% of the remains are fragmented (Table 2).

A particularly high degree of fragmentation was noted for *Mytilus* shells. The presence of both left (42.6%) and right (57.4%) valves in broadly comparable proportions (A0: 17 L and 16 R; A1: 30 L and 46 R; A2: 2 L and 4 R) indicates that mussels were likely transported to the rockshelter as complete shells (i.e., with two valves per individual). However, several mussel fragments are embedded in calcareous concretion, complicating the quantification of mussel remains to an estimate only (Fig. 4e).

**Table 2:** % of the overall integrity of the malacological assemblage across the investigated levels

	A0	A1	A1-A2	A2	A3
Complete (90–100%)	10%	41%	35.7%	35%	14%
Partially complete (> 50%)	40%	25.9%	28.6%	22.2%	46.9%
Fragmented (< 50%)	50%	33.1%	35.7%	42.8%	38.8%

##### **Decalcification**

Decalcification is a process that can affect shells in both pre-depositional (marine) and post-depositional environments (Supplementary Information 3; Fig. S3c). In marine environments, the calcium carbonate of the shell can undergo dissolution due to low temperatures and high salinity. During the early stages of decalcification, the shell starts to lose its natural sculptural features as layers of calcium and aragonite degrade. As decalcification progresses, the shell loses its original pigmentation and surface gloss, resulting in a whitish and matte appearance. In some cases, a dusty surface texture may also be observed, further indicating the extent of decalcification. A total of 346 specimens (13.1% of the sample) display signs of decalcification, with gastropods comprising 66.5% (NISP 230) and bivalves representing 29.5% (NISP 102) of the affected specimens. They were found to be distributed across all investigated levels, with notable concentrations observed in levels A1 (NISP 140; 50.5%) and A2 (NISP 126; 36.4%). The occurrence of diffuse chemical alterations suggests that the shells were exposed to pre-/post-depositional environments with particularly acidic conditions, favouring the weakening of the shell structure and accelerating the fragmentation process due to the loss of calcium carbonate.

##### Manganese staining and root etching

Manganese staining resulting in black-blue stains was observed on the internal and external surfaces of 169 specimens (6.4%). Evidence of manganese dotting and stains was documented in all levels except for A0, with the highest frequency in levels A2 (NISP 92; 54.4%) and A1 (NISP 56; 33.1%).

Root action is limited in the overall collection. In levels A1 and A2, only 14 specimens present micro-grooves attributable to plant roots and fungi. Root etching (Fig. S3d) was observed in similar amounts on both the external and internal areas of the site, indicating that comparable post-depositional processes related to root action occurred in both areas of the shelter.

#### Anthropogenic alterations

Anthropogenic modifications include burning damage, pigment residues, and human-caused modifications due to bead-making or tool production.

Perforated shells are discussed in the next section, while species with potential functional use (*Callista chione*) are presented in the supplementary material (Supplementary Information 1; Fig. S1).

##### Burning damage

Thermal alteration was recognized in the assemblage as a modification of the shells’ natural pigmentation, from lightly charred (grey) to fully calcined (white and powdery) (Claassen 1998). The presence of micro-cracks on the external surface of the shells was also recorded.

Burning damage was observed on a total of 301 remains (11.4%), with the majority concentrated in level A1. Mainly, burning damage was identified on *Mytilus* fragments. Among gastropods, the most affected species are *H. sanguineum* and *T. neritea*. Heat-altered gastropods include both unperforated (80%) and perforated shells (20%).

In level A0, heat alteration only occurs on *Mytilus* shells. Burned remains display evident signs of calcination and were exclusively distributed in the external squares (primarily squares A1 = 38.2% and B1 = 58.2%), while burnt specimens from level A3 were entirely found inside the rockshelter (squares EE1 and FF3), near the back wall.

A distribution map of levels A1 and A2, displaying different categories of plotted finds and the identified hearth structures, is included in the supplementary material (Supplementary Information 5; Fig. S5).

In level A1, the majority of the burnt shells (68.9%) were found in association with the hearth located outside the rockshelter, concentrated in square B1. *Mytilus* shells (NISP 159; 95.2%) show signs of calcination (63.1%), indicating that they underwent direct contact with the fire. Possibly, the hearth may have been used as a designated area for discarding food waste (i.e., empty valves), reflecting a certain level of site maintenance, as argued in other studies (Pothier Bouchard et al. 2020; Vallerand et al. 2024).

In level A2, charred shells were spatially concentrated both outside the rockshelter (44.7%) and in association with the internal fireplace (43.4%). The majority (NISP 29; 85.3%) of the recovered remains from the external area were identified as *Mytilus* fragments. Possibly, mussels were placed on the hot embers of the fireplace to facilitate valve opening or to roast the meat, causing darkening of the shell. After consumption, empty valves may have been discarded outside the shelter, away from the active areas. Additionally, it cannot be ruled out that some of the valves were unintentionally burned if they were previously in the area where the fire was located. Burned remains associated with the *cuvette*-type hearth features documented inside the rockshelter (squares DD1 and DD2) mainly consist of small gastropods with no nutritional value.

The analysis conducted on perforated gastropods displaying burning damage is detailed in the supplementary material (Supplementary Information 6; Fig. S6).

##### Mineral pigments

**Fig. 6:**
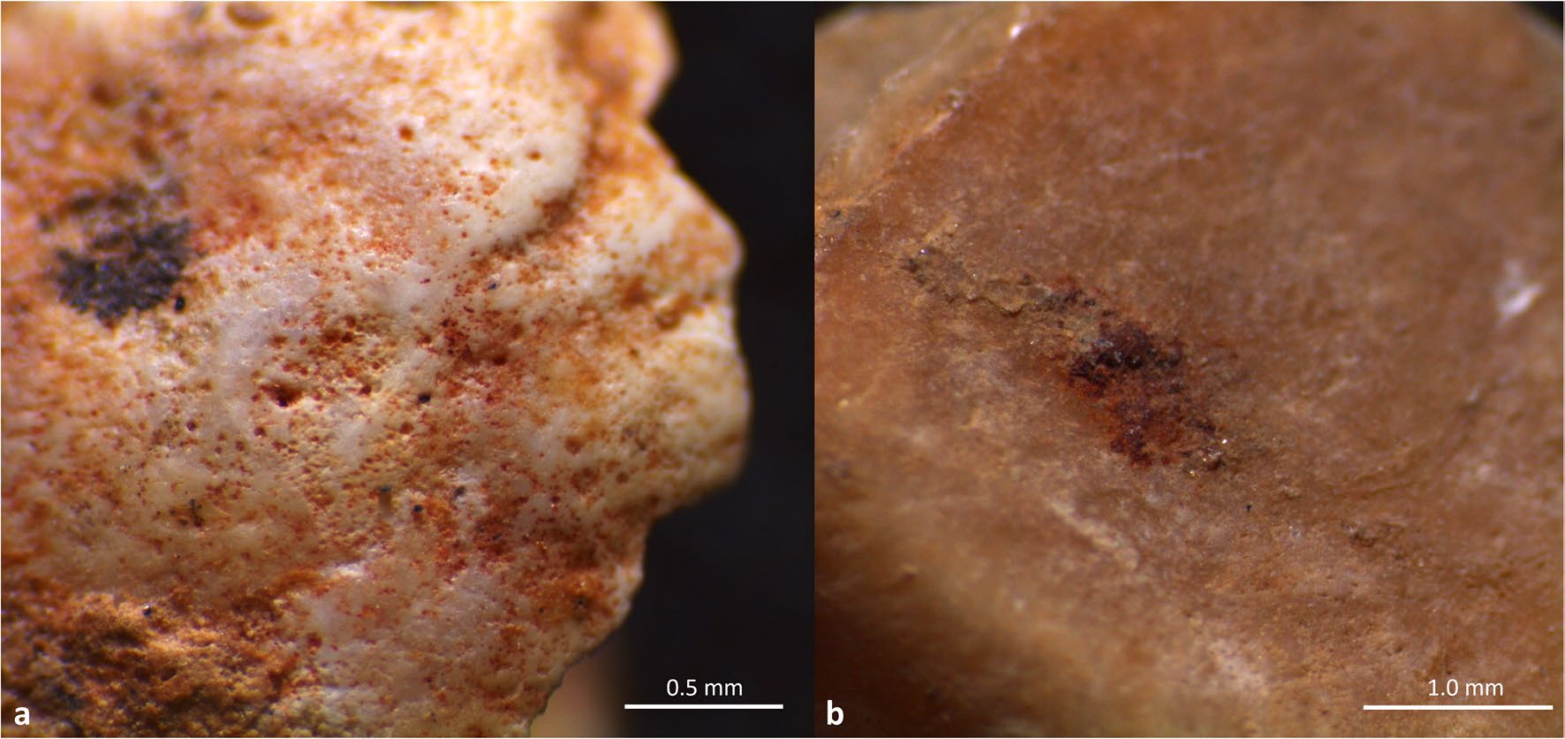
**a**) Residues of a red substance trapped inside micro-concavities of a *T. neritea* shell produced by marine bioerosion (from level A1); **b**) residues of a thick deposit of red substance inside the left valve of a *Pecten* sp. from level A1

Several shells are stained with a red pigment or bear small amounts of red residues, most likely ochre. These residues were observed on a total of 70 shell remains distributed across levels A1, A1-A2, A2 and A3; the highest frequency of ochred shells at Riparo Bombrini was found in level A2 (8.5%). The red staining primarily affects the dorsum of gastropod shells (NISP 62; 90%), while it was rarely observed on bivalves (NISP 6; 8.6%) and scaphopods (NISP 1; 1.4%). Microscopic analysis revealed traces of red pigment on a total of 29 (41.4%) perforated shells from levels A1 (NISP 16), A2 (NISP 12), and A3 (NISP 1), most of them belonging to *T. neritea* and *H. sanguineum*. The red substance, whose distribution is limited strictly to the natural ornamentation (suture and ribs) and to the inside of micro-pits produced by bioeroders, was interpreted as a pigment that was applied evenly on the outer surface of the shells (Fig. 6a). Additionally, red pigment-stained shells from levels A1 (NISP 17; 70.8%) and A2 (NISP 33; 78.6%) were widely distributed in close proximity to the hearth structures.

The presence of thicker deposits of a red substance on the internal (concave) side of a beach-polished valve of *Glycymeris* sp. (level A1-A2) and inside the left valve of *Pecten* sp. (level A1) raises the possibility that these shells served as ochre containers, something already suggested at other sites, though never before at the Balzi Rossi or in peninsular Italy (Fig. 6b) (Wadley 2015; Zilhão et al. 2010).

## Shell ornament assemblage

Shell beads have been predominantly produced using whole or partially intact marine gastropods.

**Fig. 7:**
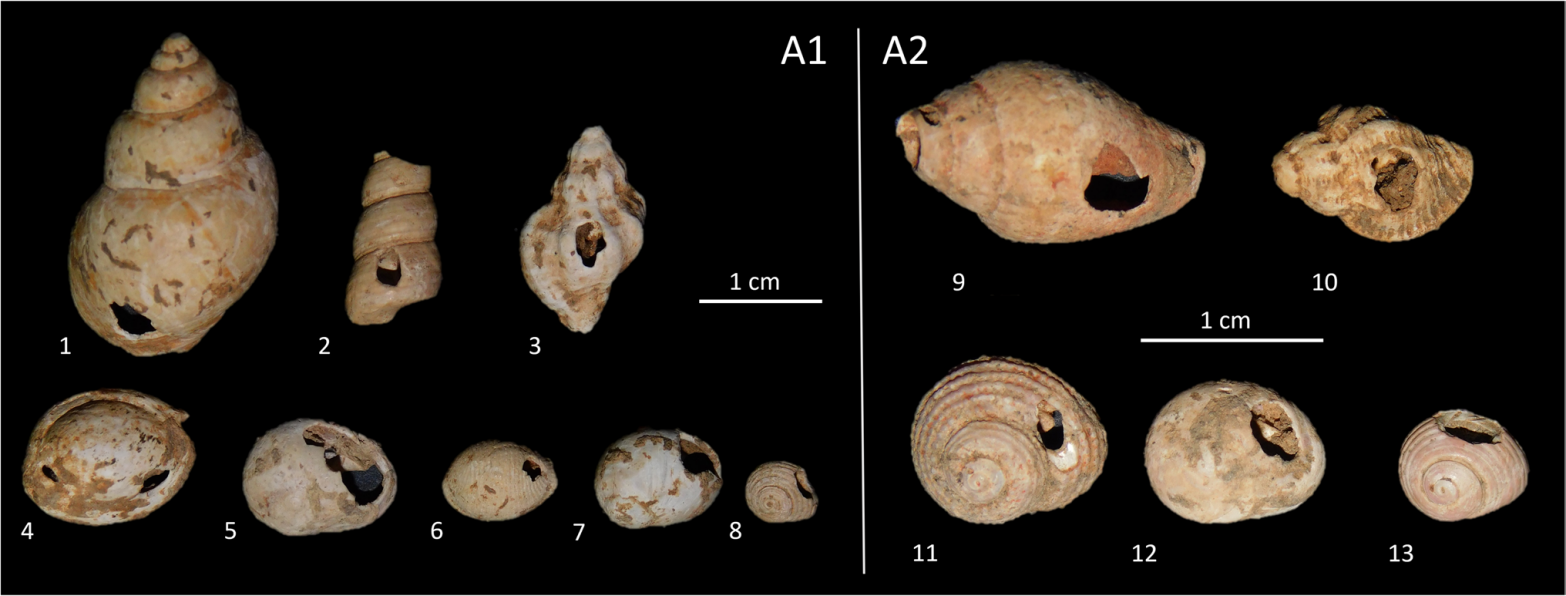
Selection of shell ornaments from levels A1 and A2. 1, *Tritia mutabilis*; 2, *Turritellinella tricarinata*; 3, *Ocenebra edwardsii*; 4, *Tritia gibbosula*; 5, *Tritia neritea*; 6, *Trivia* sp.; 7, *Euspira macilenta*; 8, *Homalopoma sanguineum*; 9, *Columbella rustica*; 10, *Ocenebra edwardsii*; 11, *Clanculus corallinus*; 12, *Tritia neritea*; 13, *Homalopoma sanguineum*

Only a small fraction (15.9%) of the collected gastropods shows evidence of human modification for ornamental purposes (Fig. 7). Among the assemblage, we identified a total of 91 perforated gastropods and four modified scaphopod fragments, while no bivalves with anthropic perforations were recorded.

The dominant taxa are *T. neritea* (NISP 26) and *H. sanguineum* (NISP 25), while other species are scarcely represented (Table 1). The rarity of certain species does not appear to be related to post-depositional processes, as their structural properties are similar to the more prevalent taxa. It is possible that less common taxa were intentionally sought out as contrasting components in ornamental elements where the more abundant species were prevalent.

Levels A1 and A2 yielded the majority of the identified perforated shells, while no ornaments were found in levels A0 and A1-A2 (Table 3).

**Table 3:** MNI and %MNI of shell ornaments from the investigated levels

SHELL ORNAMENTS		
LEVELS	MNI	%MNI
A0	-	-
A1	45	49.5
A1-A2	-	-
A2	43	47.3
A3	3	3.3
**TOTAL**	**91**	**100**

In level A1, perforated shells were distributed both inside (37.8%) and outside (62.2%) the rockshelter. Of the 19 shell beads found in squares associated with hearth features, 14 were in association with the external hearth (square B1), while five with the hearth located inside the shelter. Notably, the internal square EE2 exhibited a concentration of 14 non-perforated *H. sanguineum* shells, suggesting that this area may have served as a cache for unworked materials awaiting further modification and/or exchange.

In level A2, perforated shells were spatially concentrated in the internal area of the shelter (81.4%), mainly near the *cuvette*-type hearth feature. Additionally, one perforated *C. rustica* and one perforated *L. obtusata* s.l. were discovered in square DD1, while three small, modified fragments of *Antalis* sp. were found in squares D1 (NISP 2) and FF2 (NISP 1).

Level A3 yielded only three perforated *T. neritea* shells, distributed near the shelter wall in squares EE1 and FF3.

### Technological analysis

**Fig. 8:**
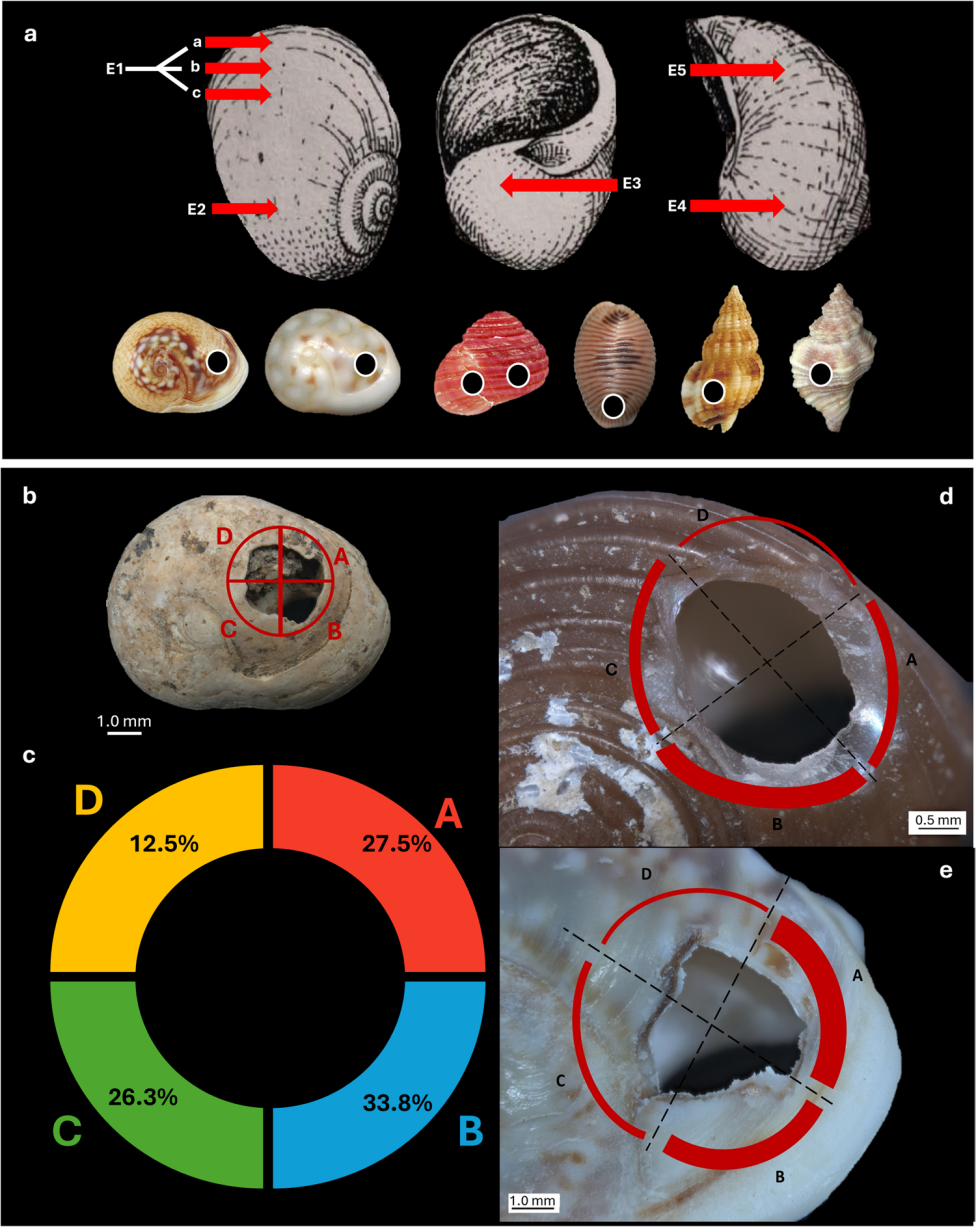
**a**) Location of anthropic perforation according to Taborin (1993a): E1, edge of the lip (E1a, very close to the edge of the lip; E1b, further away from the edge of the lip; E1c, on the back ); E1-E2, between the edge of the lip and the back; E2, base of the back; E3, under the natural aperture; E4, base of the shell (in suspension position); E5, against the columella. (modified from Taborin 1993a , p. 170). Below - location of the perforation in some of the identified taxa. From right to left: *T. neritea*, *T. pellucida*, *H. sanguineum*, *T. monacha*, *T. incrassata*, *O. edwardsii*; ** b**) Localization of the four areas identified around the rim of the shell’s perforation; **c**) graphic representation of the distribution of use wear in the four areas in all the investigated levels; **d**) graphic representation of the use wear distribution on two of the most represented species: **e**) *H. sanguineum* and **d**) *T. neritea*

Anthropic perforations occur systematically on the dorsal side of the shells, near the aperture, in correspondence of the E1 and E2 positions, according to Taborin (1993a) (Fig. 8a). No pre-boring or pecking marks were observed on the outer surface of the shells, indicating that gastropods were perforated without a precautionary preparation. Both thick and thin structured shells were perforated and later suspended as beads. Thick shells with a nacreous layer are stiff and hard (a certain controlled force is required to punch through), physical characteristics that make it a suitable material due to its long lifespan. On the other hand, shells that have a thin thickness are easier to perforate, but their fragility makes them prone to damage resulting in the hole enlargement during suspension.

Technological analysis of the perforation techniques uncovered some variability across the assemblage, notably in terms of perforation. Thick, dense shells with a nacreous layer (e.g., *H. sanguineum*) were pierced using indirect percussion through the aperture. This technique involves a single controlled motion, allowing for a better delimitation of the exact location of the hole. Perforation by pressure has to be considered unsuitable due to the need to penetrate two different layers, one of which (nacre) brings high hardness and fracture toughness to the structure (Barthelat 2016).

Considering the position of the holes on the dorsal side, the perforations might have been produced through percussion from the inside of the shells. Also, the external micro-flaking identified around the perforations is consistent with the use of internal indirect percussion. Despite the tightness of the opening, it seems to be wide enough to accommodate the insertion of a thin, pointed punch, which was possibly then struck with a pebble (i.e., hammerstone). Due to the small size and rounded shape of the shells, the use of a flat support (i.e., anvil) is required to hold the shell steady while perforating.

Experimental activity conducted on modern specimens of *H. sanguineum* has confirmed that indirect percussion on an anvil, performed through the aperture of the shell with a bone tool, is the most efficient technique for producing holes in shells of this species. The photographic documentation of the experimental activity is available in the supplementary material (Supplementary Information 7, Fig. S7).

Post-depositional damage and/or diffused use wear heavily affected some ornaments on thin structured shells, sometimes causing the fading of technological micro-chipping related to bead manufacture. For this reason, discerning the perforations technique on those species was not possible. However, drawing from previous experimental studies (e.g., Bertolini et al. 2016; Cristiani and Borić 2012), we hypothesise the use of a less forceful technique (i.e., pressure applied through the natural aperture using a wooden stick or a bone point) for shells with thin thickness such as *T. pellucida* and *T*. *neritea*. This technique aims to minimise the risk of accidental perforation-related breaks resulting from the perforation point.

**Fig. 9:**
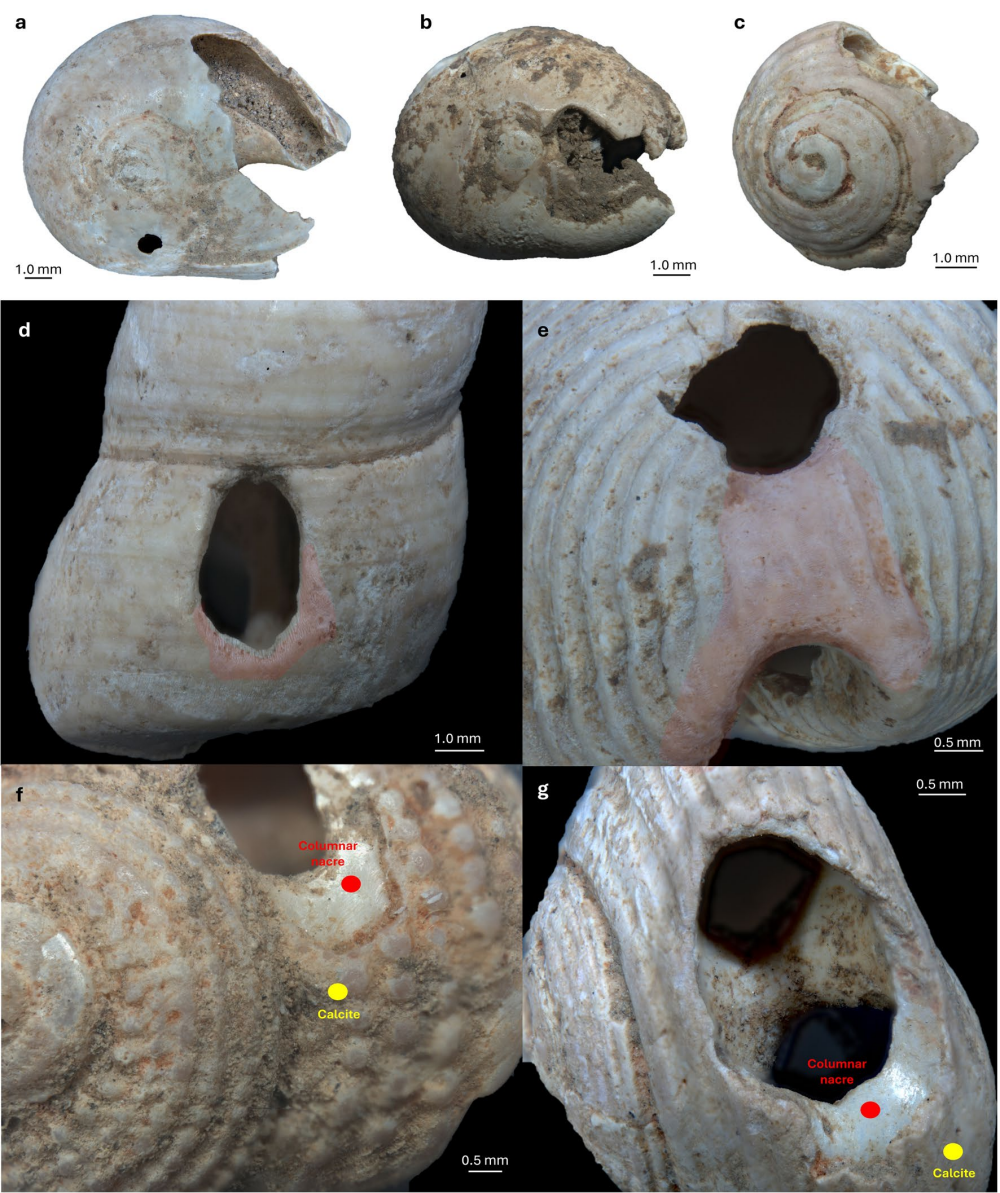
Example of manufacturing mistake (**a**) and broken perforated shells with use-wear (**b** and **c**). **a**) *T. neritea* (from level A1), the margins show a fresh appearance; **b**) *T. neritea* (from level A3), rounded features are noticeable around the rim of the perforation; **c**) *H. sanguineum* (from level A3), a slight use wear is evident around the margin of the perforation. **d**, **e**, **f** and **g**) Different deformation patterns observed on shell beads. **d** and **e**) Shells lacking a thick mother-of-pearl layer. The pink area highlights mechanical modification on the shell’s surface; **d**) rounding and flattening around the rim of the perforation of a *T. communis* from level A2; **e**) flattening and deformation of the natural ridges of the shell between the rim of the perforation and the natural aperture of a *Trivia* sp. from level A1. **f** and **g**) shells with a thick mother-of-pearl layer. On the chipping around the perforation, it is noticeable how the nacre layer has undergone a process of friction, leading to the polishing of the surface. **f**) *C. corallinus* from level A2; **g**) *H. sanguineum* from level A1

Manufacturing errors resulting in fractures were distinguished from breaks related to use by the absence of use wear on the edge of the perforation (d’Errico et al. 2005; Stiner 2010). We identified manufacturing errors on *H. sanguineum* (NISP 5) and *T. neritea* (NISP 2) shells. Five of the examined specimens display broken, crisp-edged anthropic holes with no polishes and rounding on the rim, suggesting that they were not worn but rather fractured during manufacturing (Fig. 9a). According to our experimental activity, these types of manufacturing accidents can be interpreted as the result of excessive and uncontrolled force applied during perforation. In contrast, two specimens (Fig. 9b and c) show slight use wear traces around the margin of the broken hole, suggesting ornaments breakage subsequent to their suspension.

The analysis of *Antalis* sp. (NISP 3) reveals technological modifications for the production of small tube beads. Two of the identified tusk shells have the distal portions cut off, and one of them show a rectilinear fracture associated with cut-marks – likely caused by the accidental side slipping of a lithic cutting-edge on the shell’s surface – suggesting that this operation was carried out through the ‘incision and snapping off’ technique (Supplementary Information 8; Fig. S8).

### Use wear analysis

Use wear development and distribution on perforations were examined to identify possible systems of suspension or fixation of the shell beads.

To distinguish between use wear polish and littoral abrasion, we compared texture, gloss and distribution patterns between the archaeological sample and shells from the reference collection collected from the beach. Polish from use wear is unevenly distributed on the shell, while littoral abrasion occurs uniformly on the whole surface.

Most of the observed use wear traces have a clear attritional origin, resulting from friction against a string during suspension. However, some polished surfaces may be caused by friction against fine grit, possibly from the application of red ochre. The observed use wear mainly occurs in the form of (1) polishing and rounding developed on the perforation margin (Fig. 9d), (2) flattening of the shell surface near the perforation (Fig. 9e), (3) lip deformation, and (4) notches.

Use wear on the margin of the perforation was observed on 61.5% of the analysed perforated shells.

Shell beads recorded in levels A1 and A2 exhibit localised cord-wear traces in areas A, B and C, meaning that they were oriented in a similar way during suspension. The D area, closest to the apex, is less affected by use wear, indicating less contact with the string (Fig. 8b, c, d and e). Perforated shells from level A3 show minimal use wear, suggesting rapid disposal after use.

On *H. sanguineum* shell beads and other species with a thick mother-of-pearl layer, the calcite layer and the nacreous layer respond differently to friction (García-Argudo et al. 2020) (Fig. 9f and g). Polish develops more rapidly on the outer layer of calcite, due to its fragility. In contrast, nacre exhibits flattening and polishing only on the rim of the perforation, while authentic facets and flattened/polished bands tend to develop on the calcite surface over a larger area. Among these shell beads, a total of 15 (60%) exhibit polish on both layers.

Well-defined flattened surfaces and loss of natural ornamentation concentrated in a small area between the perforation rim (A-B) and the shell aperture were primarily observed on shells with thin thickness. The presence of these specific modifications suggests that these ornaments were tied, either individually or arranged in alignment, maybe by means of a knot that occurred in this delimited area, resulting in the deformation of the calcite surface (Bonnardin 2008; Taborin 1993a).

Lip deformation (localised thinning) owing to contact with a string was observed on 19.8% (NISP 18) of the perforated shells.

**Fig. 10:**
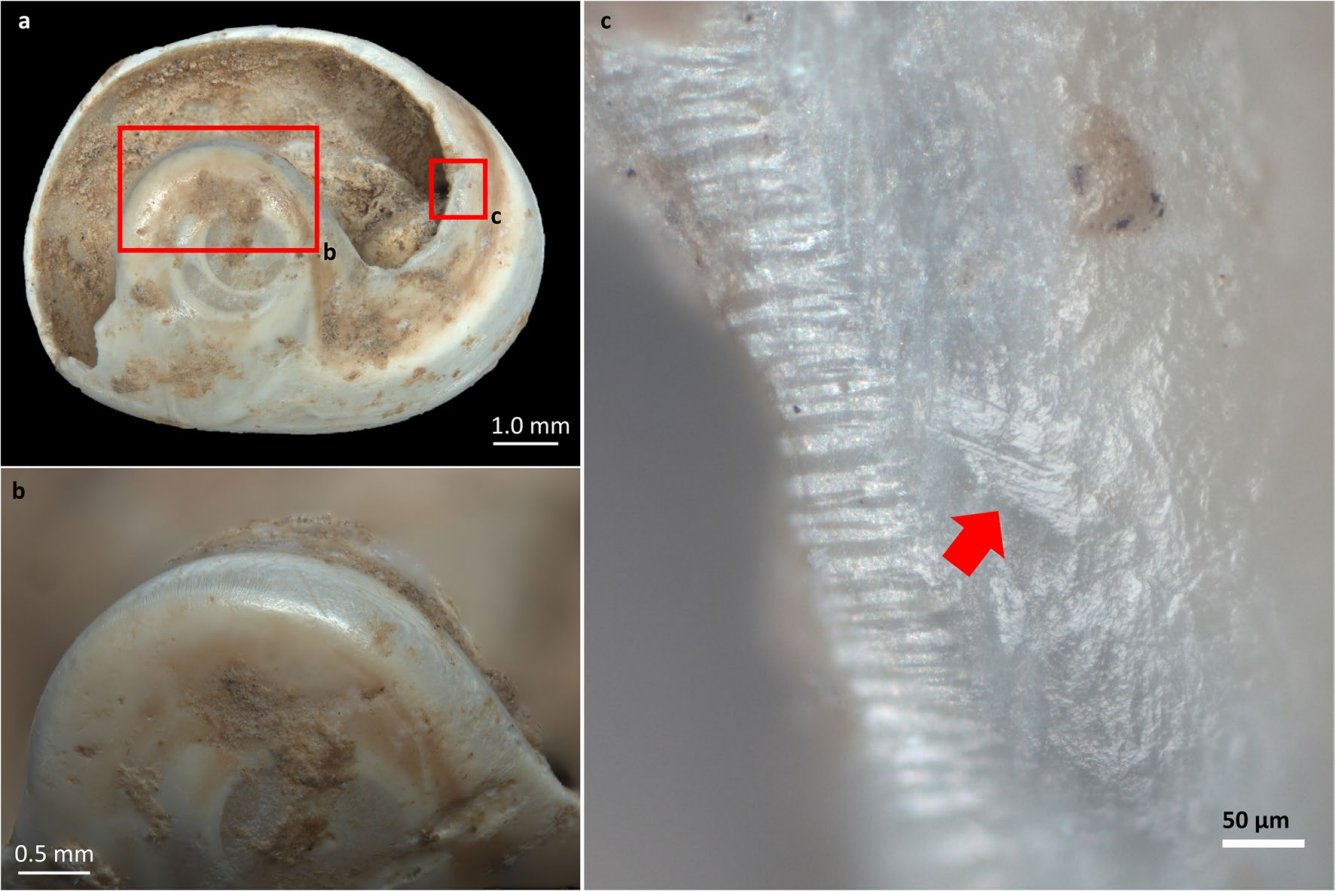
**a**) Perforated *Tritia* cfr. *neritea* shell from level A2; **b**) detail of the use wear above the apex; **c**) high-magnification view of parallel striations caused by the friction of a string along the rim of the perforation («A» position), picture taken through the ZEISS AxioScope A1 metallographic microscope (200X)

*T. pellucida/neritea* shells, which lack a nacreous layer, show more pronounced deformation of the hole (Fig. 10a and b). The observation of developed polish on the rim of enlarged perforations supports the conclusion that some *Tritia* shells were suspended for prolonged periods before being discarded or lost. Parallel striations were also observed, indicating the motion direction during suspension (Fig. 10c).

## Discussion

This study allows us to describe the Early Upper Palaeolithic coast near the Balzi Rossi area as a steep, rocky coastline associated with highly heterogeneous marine environments. These environments, with their high nutrient turnover and productivity, likely supported a rich species diversity. The Ligurian marine environment would therefore have provided a large set of mollusc species suitable for both consumption and bead manufacture, as evidenced by other Palaeolithic coastal sites located along the Ligurian basin (e.g., Stiner 1999; Taborin 1993a).

The identified shell remains can be classified into two main groups: (1) edible molluscs and (2) non-edible molluscs (Table 4). We categorised non-food specimens as those taxa lacking nutritional value due to their small size.

Generally, the shells of molluscs consumed as food and those collected for purposes other than consumption contrast sharply: the former are often extensively broken, displaying a fresh appearance with crisp, sharp edges, indicating that they were collected during life; the latter are typically wave-worn, mostly intact, and show lower incidence of burning damage (Stiner 1994, 1999, 2003).

**Table 4:** % MNI for the different shell categories identified in the studied levels

LEVELS (% MNI)	EDIBLE SPECIMENS	NON-FOOD SPECIMENS				
		Shell beads	Non-worked ornamental shells	Accidental introduction (micromolluscs)	Potential ornamental shells	Not determinable
**A0**	88.5	-	3.8	-	3.8	3.8
**A1**	23.9	17.4	27.4	16.6	10.4	4.2
**A1-A2**	15.4	-	7.7	61.5	-	15.4
**A2**	5.8	16.6	29.3	27.0	13.5	7.7
**A3**	4.8	9.5	16.7	59.5	4.8	4.8

At Riparo Bombrini, coastal resources were used for food, complementing a terrestrial diet focused on land-based protein obtained through hunting activities, mainly centred on cervids and other mammalian prey (Pothier Bouchard et al. 2020).

The malacological composition of the assemblage suggests that human groups systematically exploited the entire intertidal zone (upper, middle, and lower), in order to obtain different edible species readily available in easily accessible marine environments.

The Balzi Rossi area suffered a minor regression over time, even during the Last Glacial Maximum (LGM), when the sea level is estimated to have been about − 120 and − 140 m lower than it is today (Antonioli 2012; Benjamin et al. 2017; Lambeck et al. 2004, 2014). Analysis of the bathymetry of the seabed suggests that the shoreline was at about 2 km from the cliff during the Early Upper Palaeolithic occupations of the site (e.g., Antonioli 2012; Benjamin et al. 2017; Cerrone et al. 2021; De Lumley et al. 2001; Vacchi et al. 2018). In this regard, we must bear in mind that mollusc remains recorded on site represent only a small fraction of the molluscs that were actually consumed. It is thus likely that a larger quantity of marine resources, including shellfish, were directly consumed along the coastline, but we lack evidence of such activities since the ancient coastline is currently submerged.

Considering the environmental variability of the area, hunter-gatherers adopted diversified provisioning strategies depending on the ecology of the consumed species, aiming to optimise caloric returns and minimise time costs.

Massive consumption of marine molluscs seems to have been limited to mussels whose nutritional facts are shown in Table 5. The Mediterranean mussel form dense colonies that adhere to rocky biotypes in the intertidal zone, making them relatively easy to collect in large quantities. At Bombrini, the presence of both juvenile and adult specimens suggests that mussels were gathered *en masse*, probably in sheltered bays, without a selection of larger specimens.

**Table 5:** Nutritional values of *M. galloprovincialis* (from Santhanam 2018)

*Mytilus galloprovincialis*	
Nutritional facts (g/100 g FW)	
Total carbohydrate	1.9
Total protein	9
Total fat	1.8
Mineral	0.8
Water (%)	85.9
Dry matter (%)	13.2

Selective or ‘one by one’ procurement can be evidenced for limpets and top shells, which are found in loose clusters (Stiner 1999). While their relative rarity suggests that they made only a minor contribution to human subsistence, these molluscs were collected from the rocky substrata of the intertidal and supralittoral zones based on size criteria and not by means of an arbitrary selection, focusing on adult and large-sized individuals.

Sand dwelling and free-swimming bivalves living in subtidal and circalittoral zones (e.g., *C. chione*, *Acanthocardia* sp., *Glycymeris* sp., *P. jacobaeus*) are not documented as beads, although they were unlikely to have been used for consumption. While these species are edible with some nutritional value and are still currently exploited for their flesh along the Mediterranean coast, it seems questionable that human groups had access to these species during the molluscs’ lifespan, given the depth at which they live. The analysis of the two valves of *C*. *chione* from levels A1 and A1-A2 reveals their utilisation for non-dietary purposes in the studied archaeological assemblage.

Although there is no evidence of the use of *C. chione* as a tool in the Upper Palaeolithic of Europe, recent studies have demonstrated the use of various malacological taxa as tools in several sites attributed to Upper Palaeolithic chronologies in Europe, highlighting the significance of marine shells in the context of tools for processing ochre and other materials (e.g., Cuenca- Solana 2013, 2015; Cuenca- Solana et al. 2013, 2016).

Taxa belonging to small species with no nutritional value represent 66.3% of the assemblage. We have categorised this group into three sub-categories: (1) accidental introductions (29.1%); (2) non-worked ‘ornamental shells’ (38.2%); and (3) shell beads (32.7%).

**Fig. 11:**
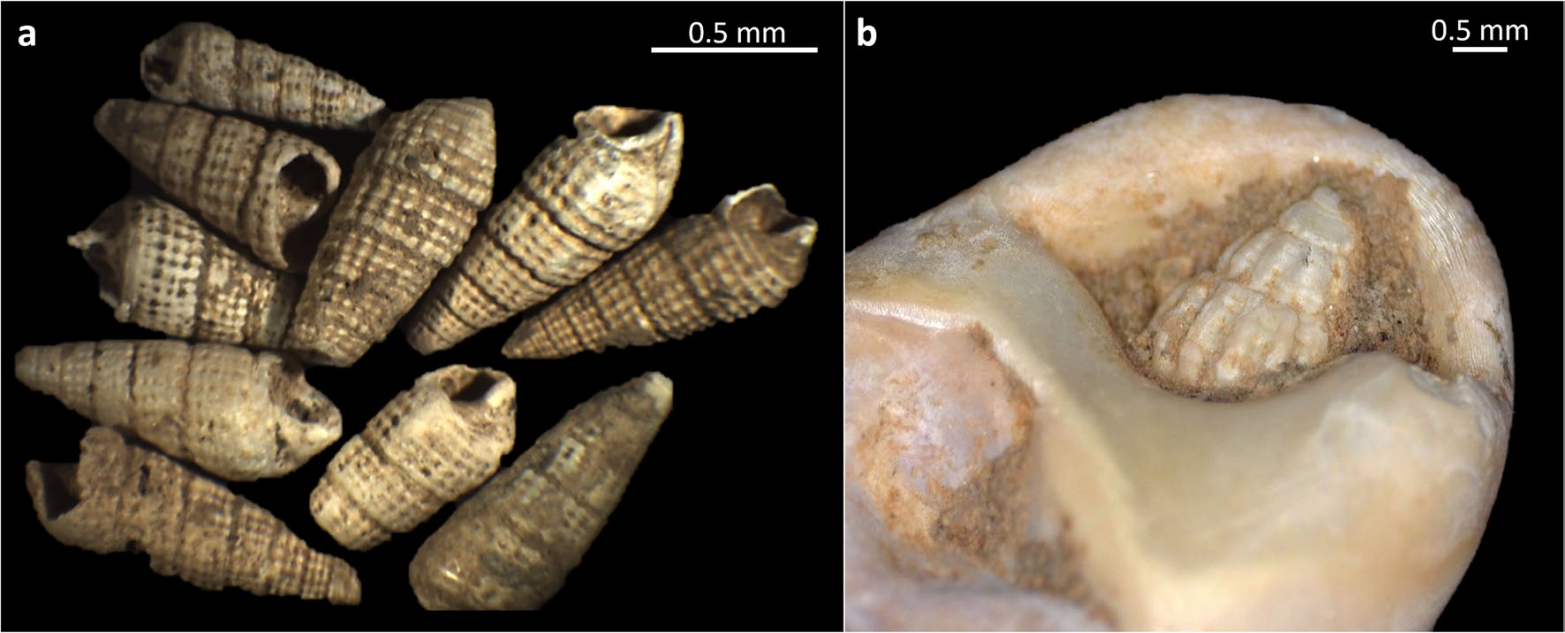
**a**) Micromolluscs (*Bittium* spp.) from Protoaurignacian levels of Riparo Bombrini; **b**) small gastropod lodged inside the natural aperture of a *T. neritea* shell (from level A2)

Micromolluscs are here considered as non-food taxa, as they are not the most appealing marine food sources due to their very small size (Fig. 11a). Natural accumulation of these shells at the site can be excluded due to the absence of specific taphonomic modifications related to birds, rodents, carnivores, or other animals that typically gather molluscs. Additionally, the distance of Riparo Bombrini from the coast makes it unlikely for geomorphological processes to be a factor in the presence of these microshells in the archaeological sediments. As such, it is likely that the micromolluscan fauna from this site do represent the remains of human activity. The questions of why and how non-dietary micromolluscs come to be deposited in anthropogenically-accumulated assemblages has puzzled archaeologists for over fifty years. Some authors have claimed that small epiphytic grazers like *Bittium* could have unintentionally reached the site, possibly mixed in with marine plants, algae and/or sponges (Barrière 1969; Colonese et al. 2018; de Lumley et al. 2004; Martini et al. 2005; Stiner 1999; Valensi et al. 2007; Verdún-Castelló and Casabó i Bernad 2020). A range of pre-depositional alterations (i.e., marine abrasion, predation, holes caused by boring sponges) observed at stereomicroscope and scanning electron microscope levels indicates that *Bittium* shells found at Riparo Bombrini reached the site post-mortem, allowing us to exclude the possibility of an introduction related to *P. oceanica* leaves, from which shells detach after the death of the mollusc. The accumulation of microshells may result from the transport of marine sediment to the site, leaving open the hypothesis of whether beach sediments might have served for specific functional activities that are still unknown, or little investigated by archaeologists (Gazzo et al. 2023).

Furthermore, it is important to consider that some micromolluscs may have reached the site along with larger shells collected from thanatocoenoses. This hypothesis is supported by the identification of very tiny shells lodged within the aperture of a total of 10 unperforated small gastropods, likely as a consequence of a prolonged contact with the beached detritus (Fig. 11b).

The ‘ornamental shells’ category includes both perforated and unperforated shells specimens. Since they fall within a type (in shape and size) that was used exclusively for ornament-making, they may represent rejected, cached, or forgotten materials that could have been potentially used as beads. It also cannot be excluded that some were collected simply out of curiosity, as an interface between the marine and the terrestrial world, for their natural origin (Τheodoropoulou 2010).

Shell modifications attributable to human activity include intentional perforations, traces that were produced by contact with a string, and ochre coloration, all of which reflect both the desire and the technological ability to suspend shell beads for display on the human body, either as personal ornaments or as decorative elements sewn onto clothing.

Apart from when they are part of ornaments recovered in burial context, beads are typically found as disconnected objects in archaeological contexts. For this reason, it is more productive, when analysing beads piece-by-piece, to think of them as the most conservative components of a flexible and more complex medium for visual communication, rather than the main objects of interest (Stiner 2013). Shell beads from Riparo Bombrini are thus considered here to be minimal components within larger, multifaceted decorative combinations (e.g., beaded caps, bracelets), which might have been lost, forgotten, rejected, and/or stored during the residential occupation of the shelter.

Damage patterns caused by marine organisms and abrasion resulting from an active beach environment indicate that ornamental shells were collected as empty shells.

Since ochre application is observed on both white and naturally red-to-vivid-pink shells, the red hues on the beads may result from the use of ochre to change the natural colour of the beads, or to enhance the colour of shells that had faded due to beach weathering. However, the possibility that the shells were rubbing against clothing, or a body smeared with ochre cannot be ruled out.

The ratio of perforated/unperforated shells at Riparo Bombrini is consistent with the general trends observed for other Early Upper Palaeolithic coastal sites such as Riparo Mochi (Balzi Rossi, Italy) (Stiner 1999) and Üçaǧizli Cave (Hatay, Turkey) (Stiner 2003; Kuhn et al. 2009). In the latter, perforation rates are very low, manufacturing errors are quite frequent, and stockpiling has been argued to have been common (Stiner 2010).

A different pattern can be observed at the inland site of Fumane Cave, in the western Lessini Mountains (North-East Italy), where a higher proportion of perforated shells was noted in the Early Upper Palaeolithic layers (Peresani et al. 2019). This evidence indicates that the farther sites are from the coast, the higher the ratio of perforated shells, suggesting a role for coastal sites as gathering and storage locations for the raw materials used in the production of shell ornaments, which agrees with the pattern highlighted at Riparo Bombrini.

Tecno-functional data demonstrate that activities related to both ornament manufacture and bead maintenance (e.g., storage of unmodified shells, discarding/replacing activities) were regularly conducted on-site. This is supported by the identification of various technological stages of the manufacturing *chaîne opératoire* (including by-products and accidental breakage that would have occurred during bead production), the presence of numerous unworked shells interpretable as a cluster or stockpile of raw material awaiting modification or trade with local or nonlocal human groups, and the presence of unused perforated shells.

Use wear analysis revealed the presence of some very worn ornaments that had been suspended for a prolonged period before being lost, intentionally discarded, or replaced in new beads. It is thus likely that the discard and replacement of broken or worn-out shell beads were regularly performed at the site, reflecting the maintenance of more complex objects of which individual shell beads were a part.

Activities related to shell selection and modification for bead production (e.g., perforation, application of ochre, replacement) were mainly concentrated inside the rockshelter, in sheltered areas associated with hearths, which also correspond to the central economic and domestic focus of the occupations.

Beads made from exotic species are very rare. If we consider the perforated *L. obtusata* s.l. shell found in level A2 as an exotic ornament of Atlantic origin, it could be interpreted as a ‘singular’ object resulting from long-distance trade routes. In this view, it is possible that such exotic items acquired a special meaning among Protoaurignacian hunter-gatherers, reflecting a perception of rarity.

## Conclusions

The data presented in this study thus establish that the Early Upper Palaeolithic occupants of Riparo Bombrini exploited marine molluscs intensively and for a variety of purposes, reflecting both human adaptations to the local coastal environment and a rather complex craft tradition.

Two different strategies of acquisition were employed: the bulk of marine resources consumed for food were collected alive from the intertidal zone, whereas shells used for bead production were collected as dead specimens from the beach shore. The rich assortment of mollusc species reflects both the diversity and stability of the Balzi Rossi coastline.

The foragers from Riparo Bombrini would have undertaken deliberate trips along the coastline in order to gather marine molluscs. While it is likely that shell collection was not their sole motivation for reaching the coast, to date there is little evidence of other marine resources at the site (e.g., fish vertebrae, sea pebbles) (Gazzo et al. 2023). Taxa having been used as food are few and their shells are found heavily fragmented; they include mussels (*M. galloprovincialis*), limpets (*Patella*) and top shells (*Phorcus*).

The exploitation of small marine gastropods and tusk shells seems to be limited to decorative purposes. Traces of manufacture, cord-wear marks and red pigment residues on the shells emphasise the symbolic use of most of these specimens at the site. On the other hand, specimens of very small-sized species, known as micromolluscs, were likely brought to the site attached to other resources from the sea.

Since shell beads are completely intermixed with lithic and bone debris in all the investigated levels, it seems that the rockshelter was used as a multi-functional site, reflecting a complex composition of the human groups, including specialised crafts-persons who made personal ornaments. From this perspective, bead manufacture is considered a complementary activity incorporated into more complex set of economic and domestic tasks.

Although the selection of ornamental species seems to be driven primarily by their natural availability in the local environment, it appears that further selections were filtered by human preferences related to aesthetic, symbolic and visual conventions, including the perception of rarity. The interest of the early AMH foragers was directed towards small, roundish, and brightly coloured species, reflecting the existence of longstanding cultural traditions shared among comparable ethno-linguistic groups (Borić and Cristiani 2019; Vanhaeren and d’Errico 2006).

Local procurement of raw material for shell bead production can be assumed given the proximity to the Tyrrhenian coast, and it seems clear that continuous cycles of production and repair took place on-site.

The probable existence of a potentially wide inter-regional exchange system between the Mediterranean and Atlantic regions is supported by the presence of two ‘cold’ species typical of the Atlantic domain (*L. obtusata* s.l. and *L. saxatilis*). However, we cannot exclude that these specimens are the result of punctual past migrations of cold-water guest in the Mediterranean. Also, it is important to note that this evidence currently stands alone. Although the presence of flint from Provence and the Rhône Valley within the Protoaurignacian lithic assemblages from Riparo Bombrini offer some support for the procurement of far-flung resources, the behaviour reflected by lithic procurement does not extend to Atlantic region, leaving the question open for the moment (Negrino et al. 2016; Negrino and Riel-Salvatore 2018; Negrino and Starnini 2003; Porraz et al. 2010).

## Supplementary Information

Below is the link to the electronic supplementary material.

ESM1 (DOCX 2.88 MB)

ESM2 (DOCX 1.01 MB)

ESM3 (DOCX 1.60 MB)

ESM4 (DOCX 19.3 KB)

ESM5 (DOCX 445 KB)  

ESM6 (DOCX 1.26 MB)

ESM7 (DOCX 4.17 MB)

ESM8 (DOCX 1.87 MB)

## Data Availability

No datasets were generated or analysed during the current study.

## Footnotes

1 The location refers to the archaeological site (Riparo Bombrini). Source: Google Earth 10.57.0.4 (2024) Riparo Bombrini, Ventimiglia (Italy). Available from: https://earth.google.com/web/ [Accessed 04 November 2024].
