# Supplementary material for: Early Upper Palaeolithic marine mollusc exploitation at Riparo Bombrini (Balzi Rossi, Italy): shellfish consumption and ornament production
Source: Archaeol Anthropol Sci. 2025 Jan 31;17(2):46. doi: 10.1007/s12520-024-02148-5 (PMC11785686; doi:10.1007/s12520-024-02148-5)
Supplement: Supplementary file 1 — (DOCX 2.88 MB) [file 12520_2024_2148_MOESM1_ESM.docx]

(Supplementary Information 1; Fig. S1)

SPECIES WITH POTENTIAL FUNCTIONAL USE (*Callista chione*)

A total of four valves of *Callista chione* showing ancient fractures from levels A1 (NISP 3) and A1-A2 (NISP 1) have been analysed. Only proximal parts, including the umbo and hinge fragments were recorded. Although *C. chione* is known for its edibility, the identification of micro-pits produced by boring sponges in the internal part of two valves suggests that these shells were collected in a dead state for non-nutritional purposes.
Two fragments of right valves found in levels A1 and A1-A2 exhibit sharp edges opposite to the umbo (Fig. S1). Similar breakage patterns have been well-documented on *C. chione* shells found in Middle Palaeolithic sites, such as Grotta dei Giganti (Cristiani and Spinapolice 2009), Grotta del Cavallo (Lecce, Italy) (Romagnoli et al. 2015), Grotta dei Moscerini (Latium Italy) (Villa et al. 2020), and Ex-Casinò (Balzi Rossi, Italy) (Oxilia 1974), where some fragments of these shells were interpreted as tools. The resistance of this species’ shell and its ergonomic morphology, facilitating a secure grip, provide the best explanation for the utilisation of these shells as a raw material for tool production during the Middle Palaeolithic (Cristiani and Spinapolice 2009; Douka and Spinapolice 2012; Oxilia 1974; Romagnoli 2015; Romagnoli et al. 2015, 2016, 2017; Villa et al. 2020). Nevertheless, no clear evidence of use wear was observed in the analyzed remains, indicating that these fragments may have been removed and discarded as waste products, as suggested for the unretouched umbos and hinges at Cavallo (Villa et al. 2020). Additionally, the absence of clear evidence of intentional breakage and the lack of other anatomical parts of the shell in the assemblage make it difficult to interpret the function of these shells at the Riparo Bombrini.


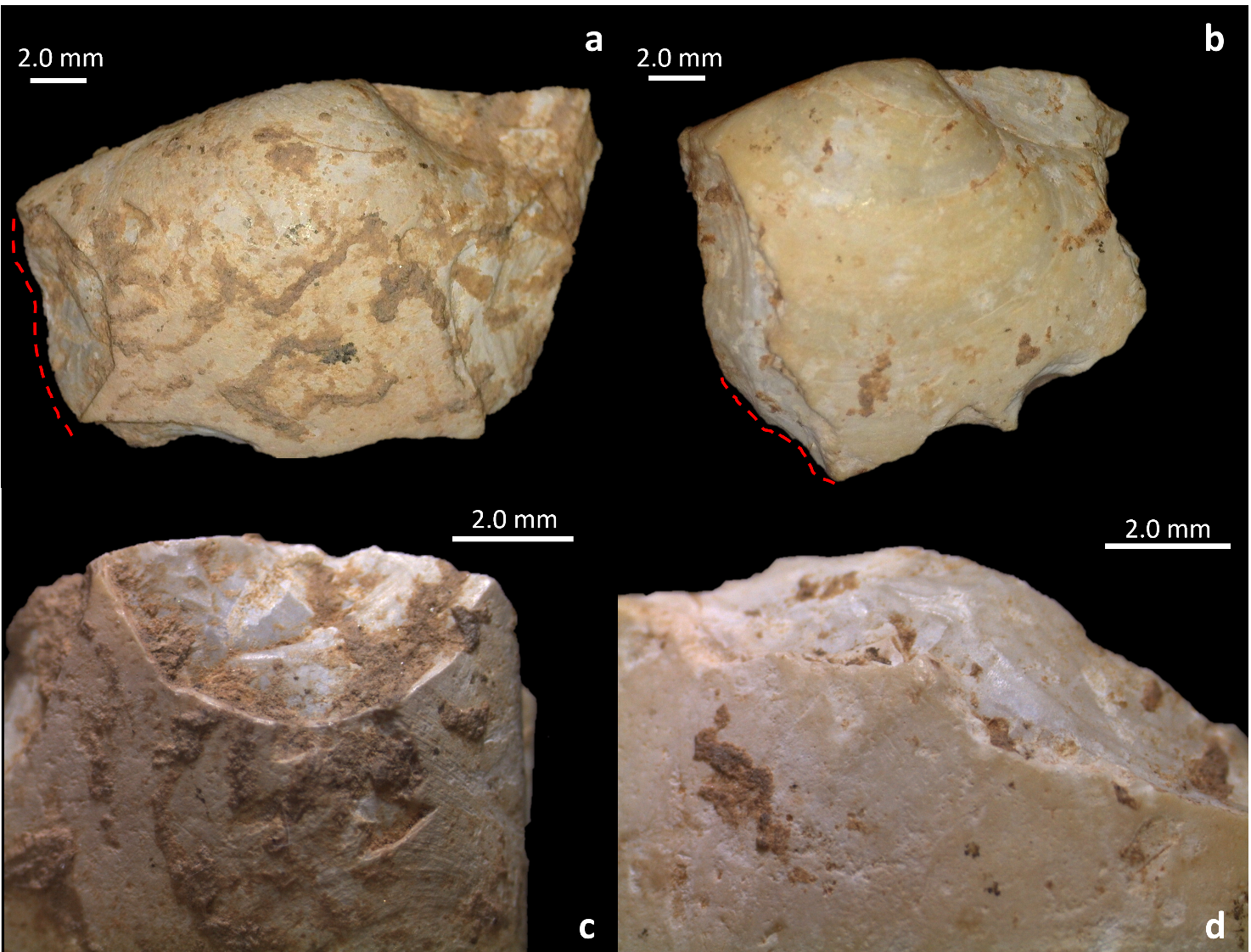


**Fig. S1** Fragments two right valves of *C. chione* from level A2 (**A** and **C**) and A1-A2 (**B** and **D**). The red dashed line in **A** and **B** indicates the portion of the shell’s margin highlighted in images **C** and **D**. The presence of sediment encrustation along the edges confirms the ancient origin of the fractures
