# Supplementary material for: Early Upper Palaeolithic marine mollusc exploitation at Riparo Bombrini (Balzi Rossi, Italy): shellfish consumption and ornament production
Source: Archaeol Anthropol Sci. 2025 Jan 31;17(2):46. doi: 10.1007/s12520-024-02148-5 (PMC11785686; doi:10.1007/s12520-024-02148-5)
Supplement: Supplementary file 2 — (DOCX 1.01 MB) [file 12520_2024_2148_MOESM2_ESM.docx]

NON-MEDITERRANEAN SPECIES

(Supplementary Information 2; Fig. S2)

Riparo Bombrini mollusc assemblage is mainly composed of species currently existing in the Mediterranean Sea, with some endemic species (i.e., *H. sanguineum* and *T. mutabilis*). However, the presence of *Littorina obtusata*/*Littorina fabalis* (NISP 2) – commonly known as flat periwinkles – and *Littorina saxatilis* (NISP 2), which are cold-water species currently absent in the Mediterranean, should be highlighted (Fig. S2).

*L. obtusata* and *L. fabalis* are two sister species widely distributed throughout the Northern Atlantic shores (Reid 1996). They are very similar in shape, colour, and general morphology, making it very difficult to distinguish them, especially when the soft diagnostic parts are not preserved (Sotelo et al. 2020; Williams 1992). We classified the flat periwinkles from Riparo Bombrini as *L. obtusata sensu lato* (s.l.) based on a combination of diagnostic traits proposed by Vanhaeren and d’Errico (2002), i.e., short spire, angled shoulder of the whorl, and slightly constricted aperture. However, it appears that specialists in *Littorina* biology agree on the near-total impossibility of distinguishing between *L. obtusata* and *L. fabalis*. *L. obtusata* is widely distributed where brown seaweeds occur, throughout the Northern Atlantic up to the Arctic Ocean shores, in European waters from Norway down to the Straits of Gibraltar and the Azores (Barkman 1955; Dautzenberg and Fischer 1914). In the Mediterranean, this species is mentioned for a few localities: Malaga, Corsica, Malta and along the coasts of Sicily. However, these finds seem to be sporadic and related to anthropic activities such as the relaying of Atlantic species (possibly *Magallana gigas*) or some attempts to give rise to a Mediterranean community of *L. obtusata* (pers. comm. E. Quaggiotto).

Biometric analysis shows that specimens of *L. obtusata* s.l. from Riparo Bombrini are significantly smaller (max. length 5.1 mm) compared to modern reference collections, as also observed by several authors for other Palaeolithic assemblages (e.g., Rigaud and Gutiérrez-Zugasti 2016; Vanhaeren and D’Errico 2002). Size difference between archaeological and modern samples may be related to both environmental conditions (e.g., water temperature and seasonal variations) and human selection (Johannesson et al. 1993; Newkirk and Doyle 1975; Queiroga et al. 2011; Reid 1993).

A similar origin can be suggested for *L. saxatilis*, common named the rough periwinkle, a species of small sea snail native to the shores of the North Atlantic Ocean, whose current presence in the Mediterranean is the result of a recent anthropogenic introduction from northern Europe. During the Pleistocene, this species lived possibly as far south as Gibraltar and the coast of Morocco (Panova et al. 2011; Reid 1996).

The presence of specimens of *L. obtusata*/*fabalis* and *L. saxatilis* in the stratigraphic complex C of Lazaret Cave (Nice, France) – associated with an Acheulean lithic assemblage – provides evidence that these species were present in the Mediterranean at least until the end of MIS 6 (Cataliotti-Valdina 1984; Valensi et al. 2007). Thus, it is conceivable that the occurrence of these taxa in the Protoaurignacian levels of Riparo Bombrini could be attributed to ancient migrations of northern taxa into the Mediterranean. However, it also cannot exclude that the recorded specimens might be the result of long-distance exchanges.


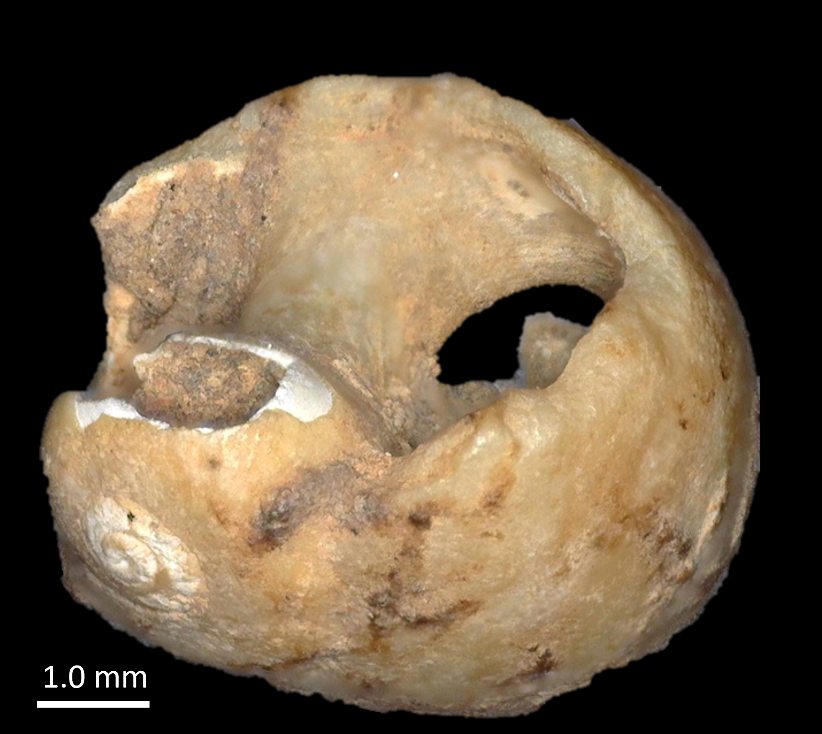


**Fig. S2** *L. obtusata* s.l. from level A2 showing an anthropic perforation with visible rounding on the margin due to prolonged use
