# Supplementary material for: Early Upper Palaeolithic marine mollusc exploitation at Riparo Bombrini (Balzi Rossi, Italy): shellfish consumption and ornament production
Source: Archaeol Anthropol Sci. 2025 Jan 31;17(2):46. doi: 10.1007/s12520-024-02148-5 (PMC11785686; doi:10.1007/s12520-024-02148-5)
Supplement: Supplementary file 3 — (DOCX 1.60 MB) [file 12520_2024_2148_MOESM3_ESM.docx]

Supplementary Information 3; Fig. S3


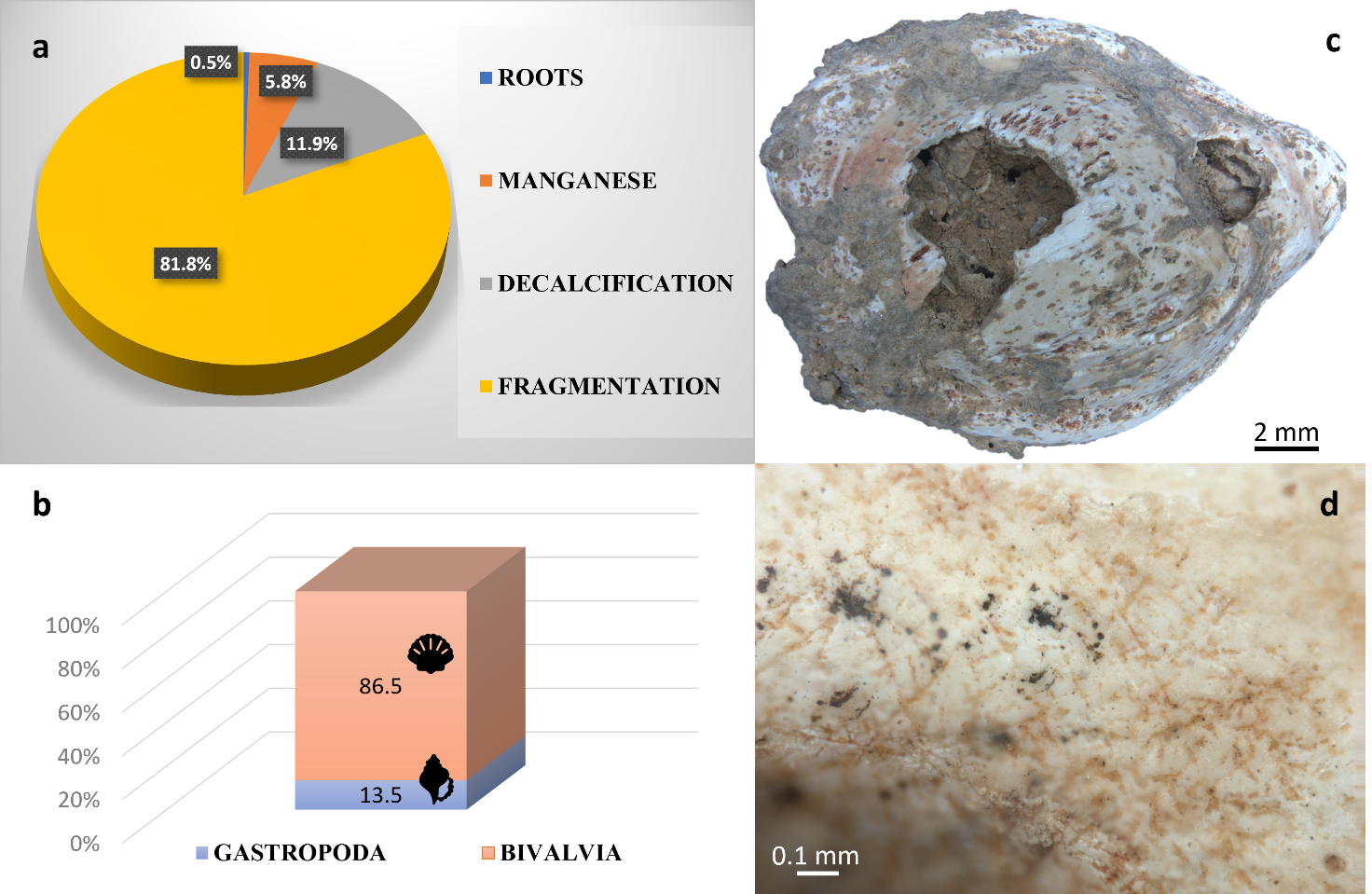


**Fig. S3** **A**) % of remains affected by post-depositional alterations (calculated on the NISP); **B**) % of fragmented bivalves and gastropods (calculated on the NISP); **C**) *T. gibbosula* affected by decalcification (from level A1); **D**) *T. neritea* affected by root etching and manganese coloration (from level A2)
