# Supplementary material for: Early Upper Palaeolithic marine mollusc exploitation at Riparo Bombrini (Balzi Rossi, Italy): shellfish consumption and ornament production
Source: Archaeol Anthropol Sci. 2025 Jan 31;17(2):46. doi: 10.1007/s12520-024-02148-5 (PMC11785686; doi:10.1007/s12520-024-02148-5)
Supplement: Supplementary file 4 — (DOCX 19.3 KB) [file 12520_2024_2148_MOESM4_ESM.docx]

Supplementary Information 4; Table S4

**Table S4** Fragmentation index (F.I.) calculated for each taxonomic family, based on the ratio MNI/NISP

| FAMILY | A0 | A1 | A1-A2 | A2 | A3 |
| --- | --- | --- | --- | --- | --- |
|  | **F.I.** | **F.I.** | **F.I.** | **F.I.** | **F.I.** |
| GASTROPODA | | | | | |
| Nassariidae | - | 1 | - | 0.9 | 0.8 |
| Cerithiidae | 1 | 0.89 | 0.89 | 0.85 | 0.96 |
| Muricidae | - | 1 | - | 0.89 | 1 |
| Trochidae | 1 | 0.92 | - | 0.81 | 0.67 |
| Littorinidae | - | 1 | 1 | 1 | - |
| Colloniidae | 1 | 0.95 | 1 | 0.94 | 1 |
| Calliostomatidae | - | 1 | - | 1 | - |
| Triviidae | - | 1 | - | 1 | - |
| Turbinidae | - | 1 | - | 1 | - |
| Columbellidae | - | 1 | - | 1 | - |
| Naticidae | - | 1 | - | 1 | - |
| Turritellidae | - | 1 | - | 1 | - |
| Rissoidae | - | - | - | 1 | - |
| Mitridae | - | - | - | 1 | - |
| Vermetidae | - | - | - | 1 | - |
| Aporrhaidae | - | - | - | 1 | - |
| Patellidae | 1 | 0.75 | - | 1 | - |
| Coralliophilidae | - | 1 | - |  | - |
| Rissoinidae | - | - | - | 1 | - |
| Conidae | - | 1 | - |  | - |
| Undetermined | - | 0.1 | - | 0.18 | - |
| TOTAL GASTROPODA | **1** | **0.91** | **0.91** | **0.84** | **0.93** |
| SCAPHOPODA | | | | | |
| Dentaliidae | - | - | - | 0.33 | 1 |
| BIVALVIA | | | | | |
| Pectinidae | 0.5 | 0.3 | - | 0.15 | - |
| Cardiidae | 1 | 0.6 | - | 0.09 | 0.5 |
| Veneridae | 1 | 0.75 | 1 | - | - |
| Glycymerididae | - | - | 1 | 0.33 | - |
| Noetiidae | - | 1 | - | 1 | - |
| Mytilidae | 0.03 | 0.04 | - | 0.03 | 0.33 |
| TOTAL BIVALVIA | **0.04** | **0.04** | **1** | **0.07** | **0.3** |
| TOTAL NISP | **0.05** | **0.17** | **0.93** | **0.54** | **0.81** |
