# Supplementary material for: Early Upper Palaeolithic marine mollusc exploitation at Riparo Bombrini (Balzi Rossi, Italy): shellfish consumption and ornament production
Source: Archaeol Anthropol Sci. 2025 Jan 31;17(2):46. doi: 10.1007/s12520-024-02148-5 (PMC11785686; doi:10.1007/s12520-024-02148-5)
Supplement: Supplementary file 5 — (DOCX 445 KB) [file 12520_2024_2148_MOESM5_ESM.docx]

Supplementary Information 5; Fig. S5

~~
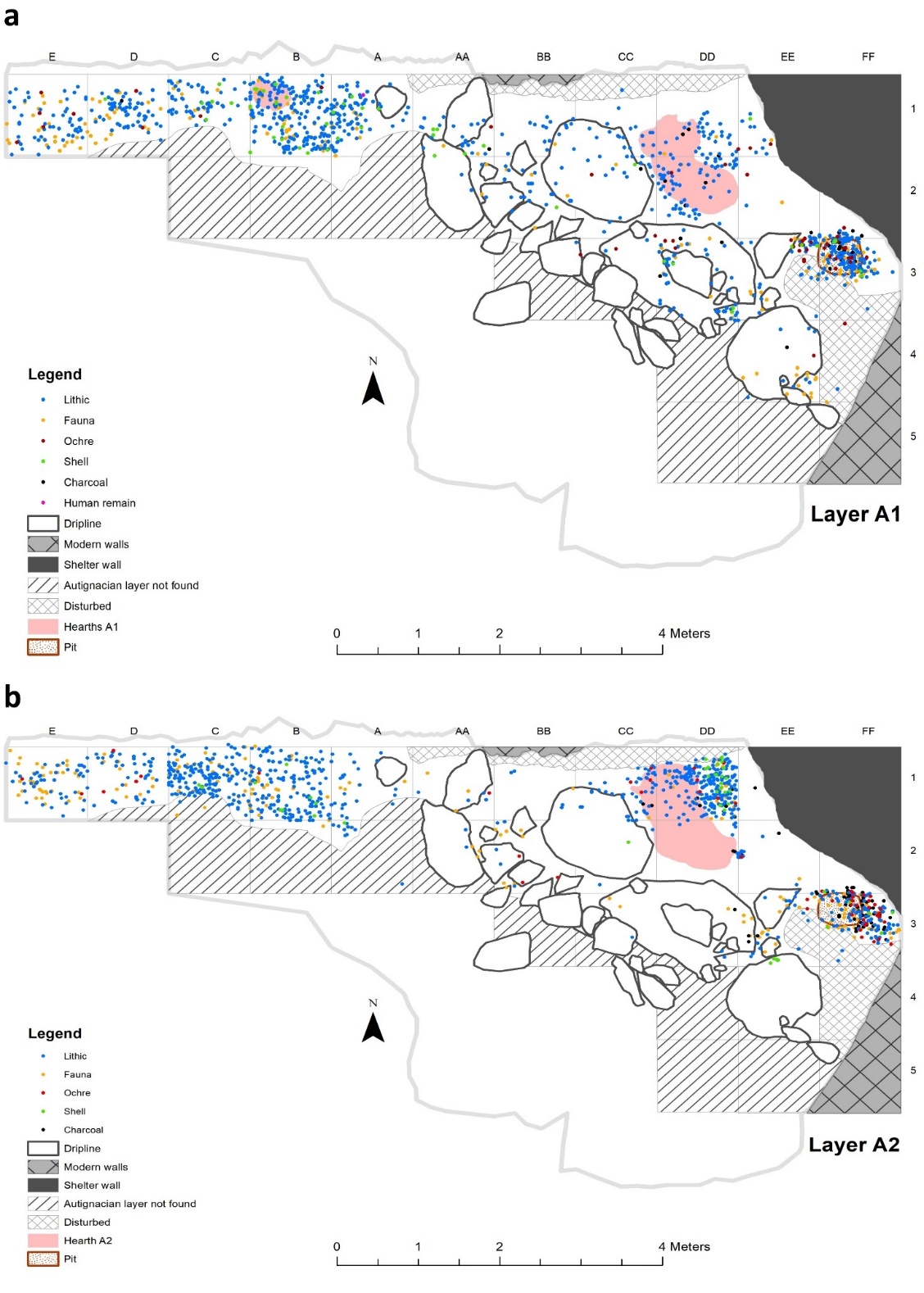
~~

**Fig. S5** Distribution map of level A1 (**A**) and A2 (**B**) showing different categories of plotted finds. Hearths identified in the two levels are highlighted in red
