# Supplementary material for: Early Upper Palaeolithic marine mollusc exploitation at Riparo Bombrini (Balzi Rossi, Italy): shellfish consumption and ornament production
Source: Archaeol Anthropol Sci. 2025 Jan 31;17(2):46. doi: 10.1007/s12520-024-02148-5 (PMC11785686; doi:10.1007/s12520-024-02148-5)
Supplement: Supplementary file 6 — (DOCX 1.26 MB) [file 12520_2024_2148_MOESM6_ESM.docx]

Supplementary Information 6; Fig. S6

BURNED SHELL BEADS

Heat-alteration was identified on perforated gastropods, specifically *T. neritea* (NISP 5; 71.4%) and *H. sanguineum* (NISP 2; 28.6%) (Fig. S6), mainly distributed in association with the recorded hearth in level A2. Considering that heating renders the shell structure more brittle (Stiner et al. 1995), and that both species can be easily perforated with a pointed tool, heat treatment for technical purposes is an unsatisfactory explanation.
The possibility of deliberate heat treatment for the purpose of altering the ornaments’ colour for aesthetic reasons was also investigated. However, this process is known to be lengthy and complex (Perlès 2019), requiring high heat and protective measures to prevent direct contact with fire or embers. Only three specimens present a shiny black coloration attributable to the accumulation of amorphous carbon, which occurs solely under reducing conditions (Lange et al. 2008). Thus, we have reason to think that the ornaments were unintentionally subjected to heat treatment, as evidenced by: (1) the limited number of blackened ornaments; (2) the coexistence of both calcined shells and bones, typically associated with direct exposure to high temperatures (i.e., flames) (Pothier Bouchard et al. 2020); and (3) the absence of blackened ornaments outside hearth placements. Moreover, the occurrence of burning damage around the perforations indicates that heating occurred after the shells were already perforated, confirming that heat alteration was accidental, as it is often the case with materials discarded in confined areas where hearths were cyclically rebuilt (Goodale 1971; Stiner et al. 1995, 2013).


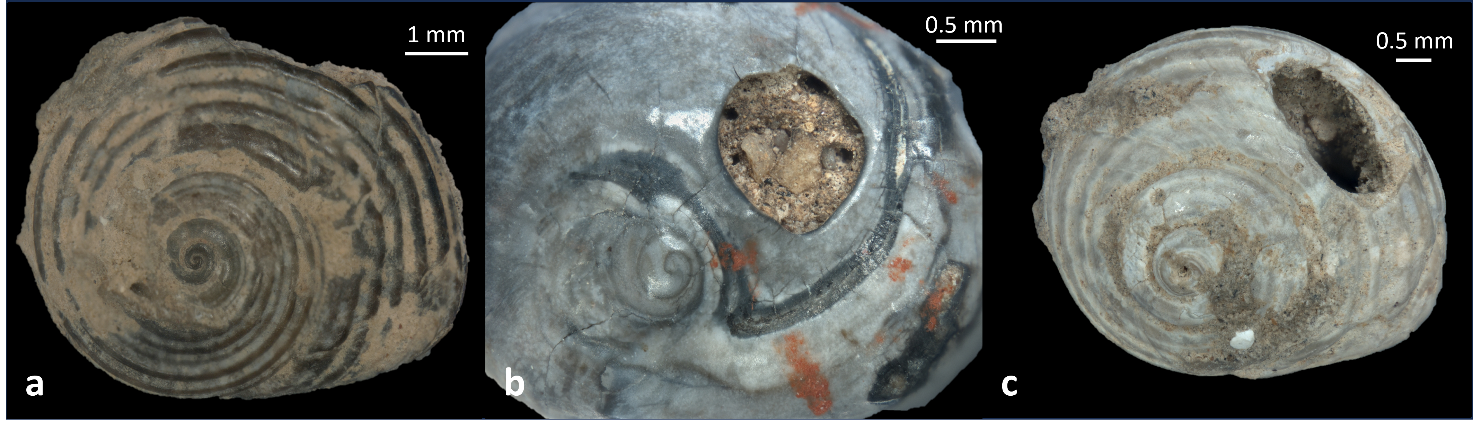


**Fig. S6** Different degrees of burning damage observed on perforated shells. **A**) *H. sanguineum* displaying a brownish coloration (from level A1); **B**) *Tritia* cfr*. neritea* exhibiting a dark grayish-black hue (from level A2); **C**) *H. sanguineum* showing a grayish-white coloration (from level A2)
