# Supplementary material for: Early Upper Palaeolithic marine mollusc exploitation at Riparo Bombrini (Balzi Rossi, Italy): shellfish consumption and ornament production
Source: Archaeol Anthropol Sci. 2025 Jan 31;17(2):46. doi: 10.1007/s12520-024-02148-5 (PMC11785686; doi:10.1007/s12520-024-02148-5)
Supplement: Supplementary file 7 — (DOCX 4.17 MB) [file 12520_2024_2148_MOESM7_ESM.docx]

Supplementary Information 7; Fig. S7

~~
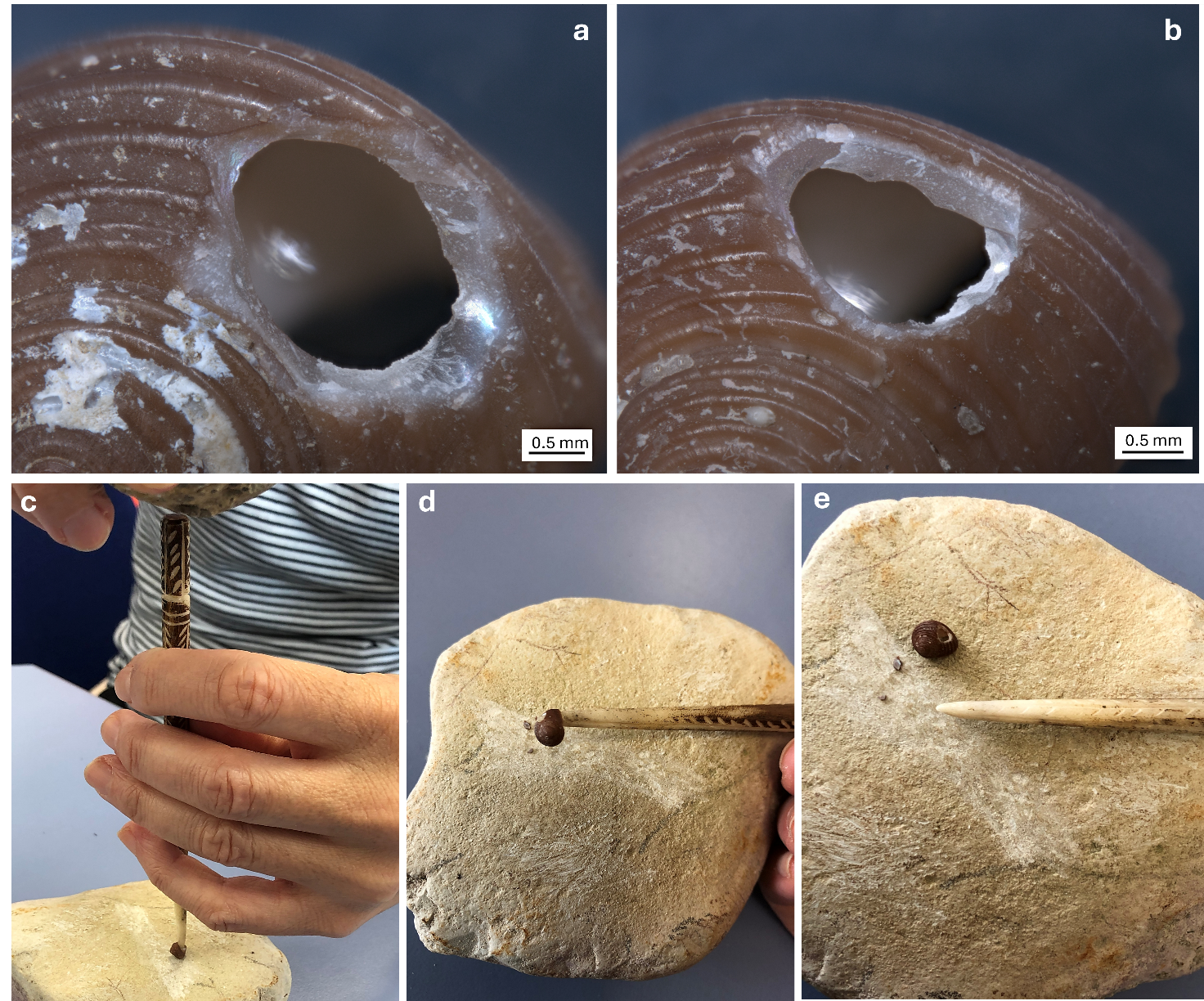
~~

**Fig. S7** A-B) Experimental perforations on *H. sanguineum* obtained by indirect percussion through the aperture of the shell. C-E) stages of experimental activity. The shell was positioned on an anvil and a pointed bone tool was placed inside the aperture of the shell; the end of the tool was then struck with a stone percussor, applying gentle force. This impact produced a hole located between E1 and E2
