# Supplementary material for: Early Upper Palaeolithic marine mollusc exploitation at Riparo Bombrini (Balzi Rossi, Italy): shellfish consumption and ornament production
Source: Archaeol Anthropol Sci. 2025 Jan 31;17(2):46. doi: 10.1007/s12520-024-02148-5 (PMC11785686; doi:10.1007/s12520-024-02148-5)
Supplement: Supplementary file 8 — (DOCX 1.87 MB) [file 12520_2024_2148_MOESM8_ESM.docx]

Supplementary Information 8; Fig. S8

~~
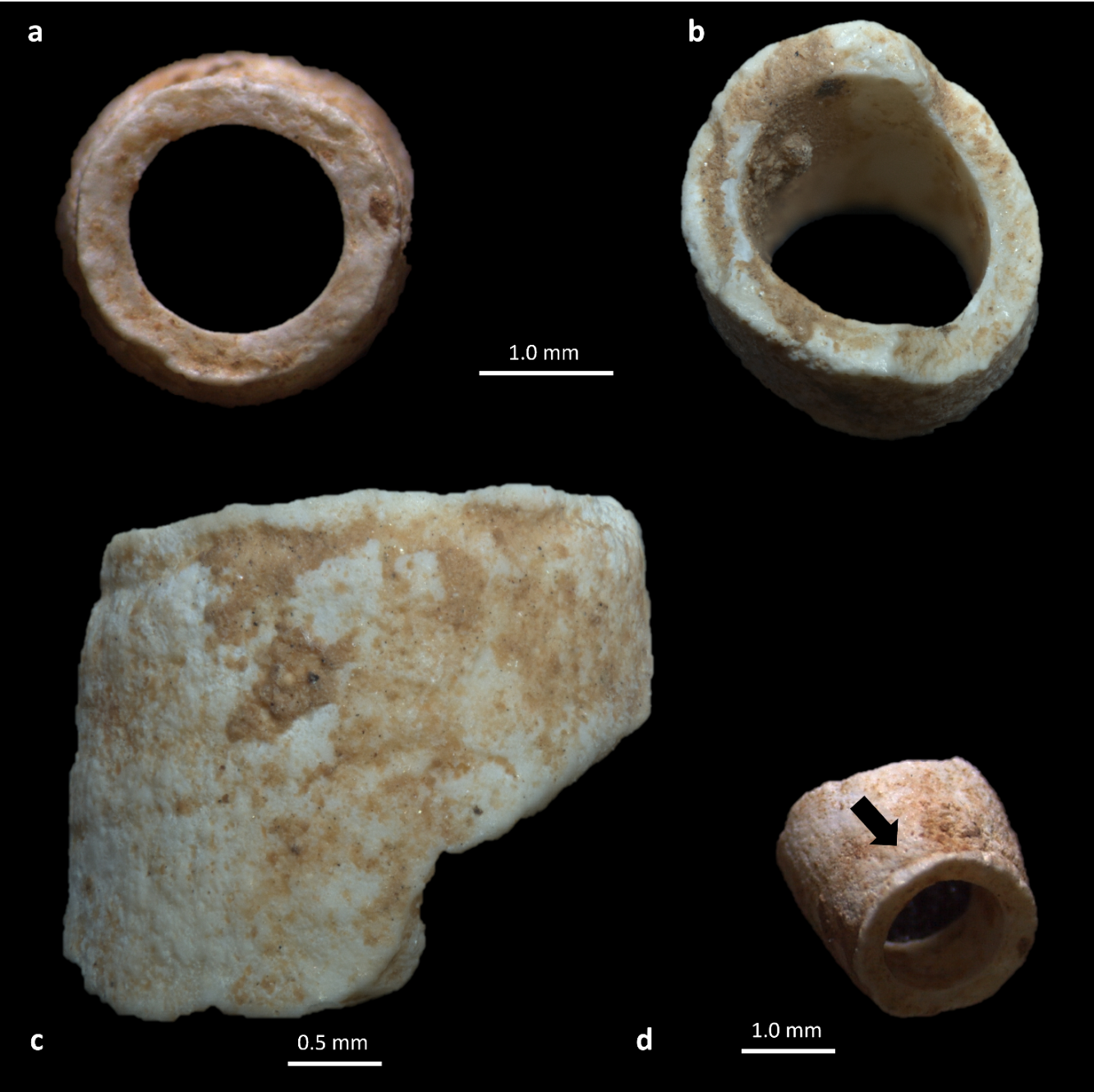
~~

**Fig. S8** Tusk shell (*Antalis* sp.) from level A3. **A**) rectilinear fracture on the apical (or distal) portion; **B**) flute-mouth fracture in the basal (or proximal) portion; **C**) front view; **D**) angled perspective, the black arrow indicates a cut-mark on the shell's surface, near the rectilinear fracture
